# A Mendelian randomization study of the role of lipoprotein subfractions in coronary artery disease

**DOI:** 10.7554/eLife.58361

**Published:** 2021-04-26

**Authors:** Qingyuan Zhao, Jingshu Wang, Zhen Miao, Nancy R Zhang, Sean Hennessy, Dylan S Small, Daniel J Rader

**Affiliations:** 1 Statistical Laboratory, University of CambridgeCambridgeUnited Kingdom; 2 Department of Statistics, University of ChicagoChicagoUnited States; 3 Perelman School of Medicine, University of PennsylvaniaPhiladelphiaUnited States; 4 Department of Statistics, University of PennsylvaniaPhiladelphiaUnited States; 5 Department of Medicine, University of PennsylvaniaPhiladelphiaUnited States; University of MelbourneAustralia; University of ZurichSwitzerland

**Keywords:** cardiocascular system, heart, blood, Human

## Abstract

Recent genetic data can offer important insights into the roles of lipoprotein subfractions and particle sizes in preventing coronary artery disease (CAD), as previous observational studies have often reported conflicting results. We used the LD score regression to estimate the genetic correlation of 77 subfraction traits with traditional lipid profile and identified 27 traits that may represent distinct genetic mechanisms. We then used Mendelian randomization (MR) to estimate the causal effect of these traits on the risk of CAD. In univariable MR, the concentration and content of medium high-density lipoprotein (HDL) particles showed a protective effect against CAD. The effect was not attenuated in multivariable analyses. Multivariable MR analyses also found that small HDL particles and smaller mean HDL particle diameter may have a protective effect. We identified four genetic markers for HDL particle size and CAD. Further investigations are needed to fully understand the role of HDL particle size.

## Introduction

Lipoprotein subfractions have been increasingly studied in epidemiological research and used in clinical practice to predict the risk of cardiovascular diseases (CVD) (Rankin et al., 2014; Mora et al., 2009; China Kadoorie Biobank Collaborative Group et al., 2018). Several studies have identified potentially novel subfraction predictors for CVD (Mora et al., 2009; Hoogeveen et al., 2014; Williams et al., 2014; Ditah et al., 2016; Lawler et al., 2017; Fischer et al., 2014) and demonstrated that the addition of subfraction measurements can significantly improve the risk prediction for CVD (Würtz et al., 2012; van Schalkwijk et al., 2014; McGarrah et al., 2016; Rankin et al., 2014). However, these observational studies often provide conflicting evidence on the precise roles of the lipoprotein subfractions. For example, while some studies suggested that small, dense low-density lipoprotein (LDL) particles may be more atherogenic (Lamarche et al., 1997; Hoogeveen et al., 2014), others found that larger LDL size is associated with higher CVD risk (Campos et al., 2001; Mora, 2009). Some recent observational studies found that the inverse association of CVD outcomes with smaller high-density lipoprotein (HDL) particles is stronger than the association with larger HDL particles (Ditah et al., 2016; Kim et al., 2016; McGarrah et al., 2016; Silbernagel et al., 2017), but other studies reached the opposite conclusion in different cohorts (Li et al., 2016; Arsenault et al., 2009). Currently, the utility of lipoprotein subfractions or particle sizes in routine clinical practice remains controversial (Superko, 2009; Mora, 2009; Davidson et al., 2011; Bays et al., 2016), as there is still a great uncertainty about their causal roles in CVD, largely due to a lack of intervention data (Bays et al., 2016).

Mendelian randomization (MR) is an useful causal inference method that avoids many common pitfalls of observational cohort studies (Smith and Ebrahim, 2003). By using genetic variation as instrumental variables, MR asks if the genetic predisposition to a higher level of the exposure (in this case, lipoprotein subfractions) is associated with higher occurrences of the disease outcome (Didelez and Sheehan, 2007). A positive association suggests a causally protective effect of the exposure if the genetic variants satisfy the instrumental variable assumptions (Didelez and Sheehan, 2007; Davey Smith and Hemani, 2014). Since MR can provide unbiased causal estimate even when there are unmeasured confounders, it is generally considered more credible than other non-randomized designs and is quickly gaining popularity in epidemiological research (Gidding et al., 2012; Davies et al., 2018). MR has been used to estimate the effect of several metabolites on CVD, but most prior studies are limited to just one or a few risk exposures at a time (Emdin et al., 2016; Ference et al., 2017).

In this study, we will use recent genetic data to investigate the roles of lipid and lipoprotein traits in the occurrence of coronary artery disease (CAD) and myocardial infarction (MI). In particular, we are interested in discovering lipoprotein subfractions that may be causal risk factors for CAD and MI in addition to the traditional lipid profile (LDL cholesterol, HDL cholesterol, and triglycerides levels). To this end, we will first estimate the genetic correlation of the lipoprotein subfractions and particle sizes with the tradition risk factors and remove the traits that have a high genetic correlation. We will then use MR to estimate the causal effects of the selected lipoprotein subfractions and particle sizes on CAD and MI. Finally, we will explore potential genetic markers for the identified lipoprotein and subfraction traits.

## Materials and methods

### GWAS summary datasets and lipoprotein particle measurements

Table 1 describes all GWAS summary datasets used in this study, including two GWAS of the traditional lipid risk factors (Willer et al., 2013; Hoffmann et al., 2018), two recent GWAS of the human lipidome (Kettunen et al., 2016; Davis et al., 2017), and three GWAS of CAD or MI (Nikpay et al., 2015; Nelson et al., 2017; Abbott et al., 2018). In the two GWAS of the lipidome (Kettunen et al., 2016; Davis et al., 2017), high-throughput nuclear magnetic resonance (NMR) spectroscopy was used to measure the circulating lipid and lipoprotein traits (Soininen et al., 2009). We investigated the 82 lipid and lipoprotein traits measured in these studies that are related to very-low-density lipoprotein (VLDL), LDL, intermediate-density lipoprotein (IDL), and HDL subfractions and particle sizes. All the subfraction traits are named with three components that are separated by hyphens: the first component indicates the size (XS, S, M, L, XL, XXL); the second component indicates the fraction according to the lipoprotein density (VLDL, LDL, IDL, HDL); the third component indicates the measurement (C for total cholesterol, CE for cholesterol esters, FC for free cholesterol, L for total lipids, P for particle concentration, PL for phospholipids, TG for triglycerides). For example, M-HDL-P refers to the concentration of medium HDL particles.

**Table 1.** Information about the GWAS summary datasets used in this article. The columns are the phenotypes reported by the GWAS studies, the consortium or name of the first author of the publication, PubMed ID, population, sample size, other GWAS datasets with other lapping sample, and URLs we used to download the datasets.

Phenotype	Dataset name	PubMed ID	Population	Sample size	Sample overlap with other datasets	URL to summary dataset
Traditional lipid traits	GERA	29507422 Hoffmann et al., 2018	Multi-ethnic	94,674		ftp://ftp.ebi.ac.uk/pub/databases/gwas/summary_statistics/
	GLGC	24097068 Willer et al., 2013	European	188,578	Kettunen, CARDIoGRAMplusC4D	http://csg.sph.umich.edu/abecasis/public/lipids2013/
Lipoprotein subfraction traits	Davis	29084231 Davis et al., 2017	Finnish	8372		http://csg.sph.umich.edu/boehnke/public/metsim-2017-lipoproteins/
	Kettunen	27005778 Kettunen et al., 2016	European	24,925	GLGC, CARDIoGRAMplusC4D	http://www.computationalmedicine.fi/data#NMR_GWAS
Heart disease traits	CARDIoGRAMplusC4D (CAD)	26343387 Nikpay et al., 2015	Mostly European	185,000	GLGC, Kettunen	http://www.cardiogramplusc4d.org/data-downloads/
	CARDIoGRAMplusC4D + UK Biobank (CAD)	28714975 Nelson et al., 2017	Mostly European			
	UK Biobank (MI)	Interim round two release Abbott et al., 2018	European	360,420		http://www.nealelab.is/uk-biobank/

Aside from the concentration and content of lipoprotein subfractions, the two lipidome GWAS also measured the traditional lipid traits (TG, LDL-C, HDL-C), the average diameter of the fractions (VLDL-D, LDL-D, HDL-D) and the concentration of apolipoprotein A1 (ApoA1) and apolipoprotein B (ApoB). A full list of the lipoprotein measurements investigated in this article can be found in Appendix 1.

### Genetic correlation and phenotypic screening

Genetic correlation is a measure of association between the genetic determinants of two phenotypes. It is conceptually different from epidemiological correlation that can be directly estimated from cross-sectional data. In this study, we applied the LD-score regression (Bulik-Sullivan et al., 2015) to the lipidome GWAS (Kettunen et al., 2016; Davis et al., 2017) to estimate the genetic correlations between the lipoprotein subfractions, particle sizes, and traditional risk factors. We then removed lipoprotein subfractions and particle sizes that are strongly correlated with the traditional risk factors, defined as an estimated genetic correlation > 0.8 with TG, LDL-C, HDL-C, ApoB, or ApoA1 in the GWAS published by Davis et al., 2017. Because these traits are largely co-determined with the traditional risk factors, they do not represent independent biological mechanisms and may lead to multicollinearity issues in multivariate MR analyses. Finally, we obtained an independent estimate of the genetic correlations between the selected traits by applying the LD score regression to the GWAS published by Kettunen et al., 2016. We used Bonferroni's procedure to correct for multiple testing (familywise error rate at 0.05).

### Three-sample Mendelian randomization design

For MR, we employed a three-sample design (Zhao et al., 2019b) in which one GWAS was used to select independent genetic instruments that are associated with one or several lipoprotein measures. The other two GWAS were then used to obtain summary associations of the selected SNPs with the exposure and the outcome, as in a typical two-sample MR design (Pierce and Burgess, 2013; Hemani et al., 2016). More specifically, the selection GWAS was used to create a set of SNPs that are in linkage equilibrium with each other in a reference panel (distance >10 megabase pairs, $r^{2}<0.001$). This was done by ordering the SNPs by the p-values of their association with the trait(s) under investigation and then selecting them greedily using the linkage-disequilibrium (LD) clumping function in the PLINK software package (Purcell et al., 2007). To avoid winner's curse, we require the other two GWAS to have no overlapping sample with the selection GWAS.

As the GWAS published by Davis et al., 2017 has a smaller sample size, we used it to select the genetic instruments so the larger dataset can be used for statistical estimation. In univariable MR, associations of the selected SNPs with the exposure trait (a lipoprotein subfraction or a particle size trait) were obtained from the GWAS published by Kettunen et al., 2016 and the associations with MI were obtained using summary data from an interim release of UK BioBank (Abbott et al., 2018). To maximize the statistical power, we used the so-called ‘genome-wide MR’ design. Independent SNPs are selected by using LD clumping, but we do not truncate the list of SNPs by their p-values. More details about this design can be found in a previous methodological article (Zhao et al., 2019b).

To control for potential pleiotropic effects via the traditional risk factors, we performed two multivariable MR analyses for each lipoprotein subfraction or particle size under investigation. The first multivariable MR analysis considers four exposures: TG, LDL-C, HDL-C, and the lipoprotein measurement under investigation. The second multivariable MR analysis replaces LDL-C and HDL-C with ApoB and ApoA1, in accordance with some recent studies (Richardson et al., 2020). SNPs were ranked by their minimum p-values with the four exposures and are selected as instruments only if they were associated with at least one of the four exposures (p-value $\leq 10^{-4}$). Both multivariable MR analyses used the Davis (Davis et al., 2017) and GERA (Hoffmann et al., 2018) datasets for instrument selection, the Kettunen (Kettunen et al., 2016) and GLGC (Willer et al., 2013) datasets for the associations of the instruments with the exposures, and the CARDIoGRAMplusC4D + UK Biobank (Nelson et al., 2017) dataset for the associations with CAD.

### Statistical estimation

For univariable MR, we used the robust adjusted profile score (RAPS) because it is more efficient and robust than many conventional methods (Zhao et al., 2020; Zhao et al., 2019b). RAPS can consistently estimate the causal effect even when some of the genetic variants violate instrumental variables assumptions. For multivariable MR, we used an extension to RAPS called GRAPPLE to obtain the causal effect estimates of multiple exposures (Wang et al., 2020). GRAPPLE also allows the exposure GWAS to have overlapping sample with the outcome GWAS, while the original RAPS does not. We assessed the strength of the instruments using the modified Cochran's Q statistic (Sanderson et al., 2019). Because many lipoprotein subfraction traits were analyzed simultaneously, we used the Benjamini-Hochberg procedure to correct for multiple testing (Benjamini and Hochberg, 1995) and the false discovery rate was set to be 0.05. More detail about the statistical methods can be found in Appendix 3.

**Table 2.** Results of some multivariable Mendelian randomization analyses. Each row in the table corresponds to a multivariable MR analysis with traditional lipid profile and the specified lipoprotein subfraction or particle size trait. Reported numbers are the point estimates and 95% confidence intervals of the exposure effect.

Trait	Effect of TG	Effect of LDL-C	Effect of HDL-C	Effect of subfraction/particle size
None	0.19 [0.09,0.29]	0.38 [0.33,0.44]	−0.053 [-0.13,0.03]	
M-HDL-P	0.37 [0.22,0.52]	0.39 [0.32,0.45]	0.30 [0.08,0.52]	−0.69 [-1.09,–0.3]
S-HDL-P	0.23 [0.12,0.33]	0.45 [0.38,0.52]	−0.11 [-0.2,–0.02]	−0.33 [-0.52,–0.15]
HDL-D	0.11 [0.00,0.22]	0.42 [0.36,0.49]	−0.44 [-0.69,–0.2]	0.33 [0.11,0.56]
	Effect of TG	Effect of ApoB	Effect of ApoA1	Effect of Subfraction/Particle size
None	0.05 [-0.05,0.14]	0.49 [0.38,0.60]	−0.095 [-0.21,0.02]	
M-HDL-P	−0.00 [-0.18,0.17]	0.50 [0.31,0.69]	0.13 [-0.06,0.32]	−0.47 [-0.80,–0.15]
S-HDL-P	0.07 [-0.03,0.17]	0.53 [0.41,0.65]	−0.13 [-0.25,–0.02]	−0.24 [-0.40,–0.08]
HDL-D	0.06 [-0.04,0.15]	0.61 [0.47,0.76]	−0.46 [-0.73,–0.19]	0.30 [0.08,0.52]

### Genetic markers for lipoprotein subfractions and CAD

To obtain genetic markers, we selected SNPs that are associated with the lipoprotein measurements identified in the MR (p-value $\leq 5\times 10^{-8}$) and CAD (p-value ≤0.05) but are not associated with LDL-C or ApoB (p-value $\geq 10^{-3}$). To maximize the power of this exploratory analysis, we meta-analyzed the results of the two lipidome GWAS (Kettunen et al., 2016; Davis et al., 2017) by inverse-variance weighting. For the associations with LDL-C and CAD, we used the GWAS summary data reported by the GLGC (Willer et al., 2013) and CARDIoGRAMplusC4D (Nelson et al., 2017) consortia. We used LD clumping to obtain independent markers (Purcell et al., 2007) and then validate the markers using tissue-specific gene expression data from the GTEx project.

### Sensitivity analysis and replicability

Because we had multiple GWAS summary datasets for the lipoprotein subfractions and CAD/MI (Table 1), we swapped the roles of the GWAS datasets in the three-sample MR design whenever permitted by the statistical methods to obtain multiple statistical estimates. These estimates are not completely independent of the primary results, but they can nonetheless be used to assess replicability. As a sensitivity analysis, We further analyzed univariable MR using inverse-variance weighting (IVW) (Burgess et al., 2013) and weighted median (Bowden et al., 2016) and compared with the primary results obtained by RAPS. We also assessed the assumptions made by RAPS using some diagnostic plots suggested in previous methodological articles (Zhao et al., 2019b).

## Results

### Genetic correlations and phenotypic screening

We obtained the genetic correlations of the lipoprotein subfractions and particle sizes with the traditional lipid risk factors: TG, LDL-C, HDL-C, ApoB, and ApoA1 (Table 1). We found that almost all VLDL subfractions traits (besides those related to very small VLDL subfraction) and the mean VLDL particle diameter have an estimated genetic correlation with TG very close to 1. Most traits related to the large and very large HDL subfractions also have a high genetic correlation with HDL-C and ApoA1.

**Figure 1.**
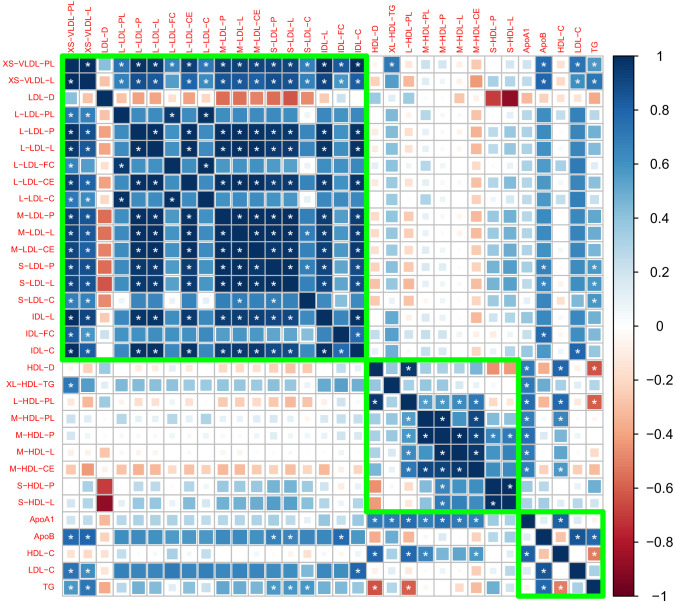
Genetic correlation matrix of the 27 lipoprotein subfraction traits selected in phenotypic screening and five traditional lipid traits. White asterisk indicates the correlation is statistically significant after Bonferroni correction for multiple comparisons at level 0.05.

After removing traits that are strongly correlated with the traditional risk factors, we obtained 27 traits that may involve independent genetic mechanisms. Figure 1 shows the genetic correlation matrix for these traits and the traditional lipid factors. The selected traits can be divided into two groups based on whether they are related to VLDL/LDL/IDL particles or HDL particles. Within each group, most traits were strongly correlated with the others. In the first group, most traits had a positive genetic correlation with LDL-C and ApoB, while in the second group, most traits had a positive genetic correlation with HDL-C and ApoA1. Exceptions include LDL-D, which had a negative but statistically non-significant genetic correlation with LDL-C and ApoB, and S-HDL-P and S-HDL-L, which showed no or weak genetic correlation with HDL-C and ApoA1.

### Mendelian randomization

**Figure 2.**
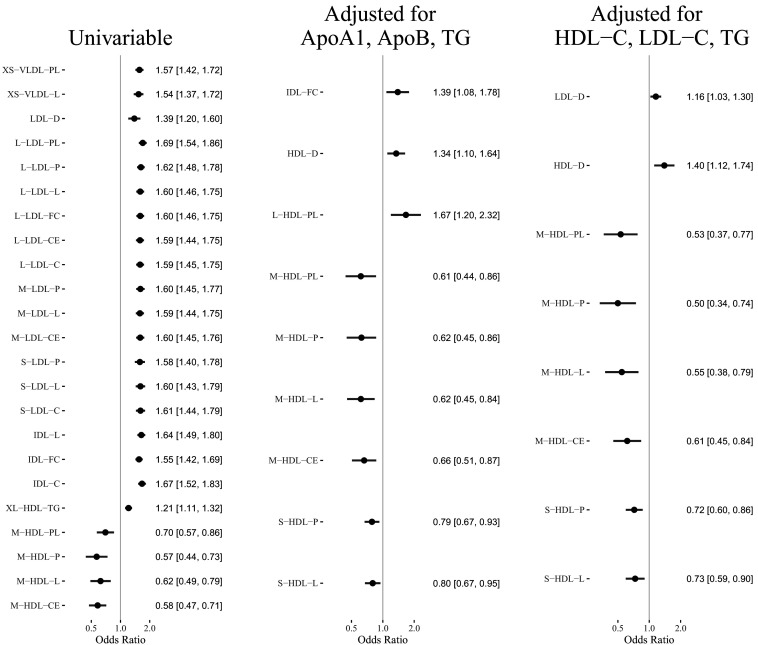
Results of the Mendelian randomization analyses (false discover rate = 0.05): Estimated odds ratio [95% confidence interval] per standard deviation increase of the selected lipoprotein measurements on MI or CAD.

Figure 2 shows the estimated causal effect of the selected lipoprotein measurements on MI or CAD that are statistically significant (false discovery rate = 0.05). The unfiltered results can be found in Appendix 3, which also contains results of the sensitivity and replicability analyses.

The concentration and lipid content of VLDL, LDL, and IDL subfractions showed harmful and nearly uniform effects on MI in univariable MR. However, after adjusting for the traditional lipid risk factors, the effects of these ApoB-related subfractions become close to zero (besides IDL-FC in one multivariable analysis). The mean diameter of LDL particles (LDL-D) showed a harmful effect on MI in univariable MR, though the effect was smaller than those of the LDL subfractions in univariable MR. The estimated effect of LDL-D was attenuated in the multivariable MR analyses.

The concentration and content of medium HDL particles showed protective effects in univariable and multivariable MR analyses. In particular, adjusting for the traditional lipid risk factors did not attenuate the effect of traits related to medium HDL. The concentration of and total lipid in small HDL particles showed protective effects in multivariable MR analyses, though the effect sizes were smaller than those of the medium HDL traits. The mean diameter of HDL particles (HDL-D) had almost no effect on MI in the univariable MR analysis, but after adjusting for the traditional lipid risk factors, it showed a harmful effect.

Table 2 reports the estimated effects of M-HDL-P, S-HDL-P, HDL-D, and traditional lipid traits (TG, LDL-C, HDL-C, ApoB, ApoA1) in the multivariable MR analyses. To better understand the role of HDL subfractions and particle sizes, we also included in the table the results of the multivariate MR analyses for the traditional lipid risk factors only. Those baseline analyses suggested that HDL-C/ApoA1 had a weak, non-significant protective effect on CAD, which is consistent with prior studies (Holmes et al., 2015; Wang et al., 2020). Adding S-HDL-P to the MR analysis did not substantially alter the estimated effects of the traditional lipid traits. However, when M-HDL-P or HDL-D was included in the model, the estimated effects of M-HDL-P and HDL-D changed substantially. In particular, when M-HDL-P was included in the multivariable MR analyses, HDL-C/ApoA1 showed a harmful effect on CAD. When HDL-D was included, HDL-C/ApoA1 showed a protective effect.

### Genetic markers associated with HDL subfractions and CAD

**Figure 3.**
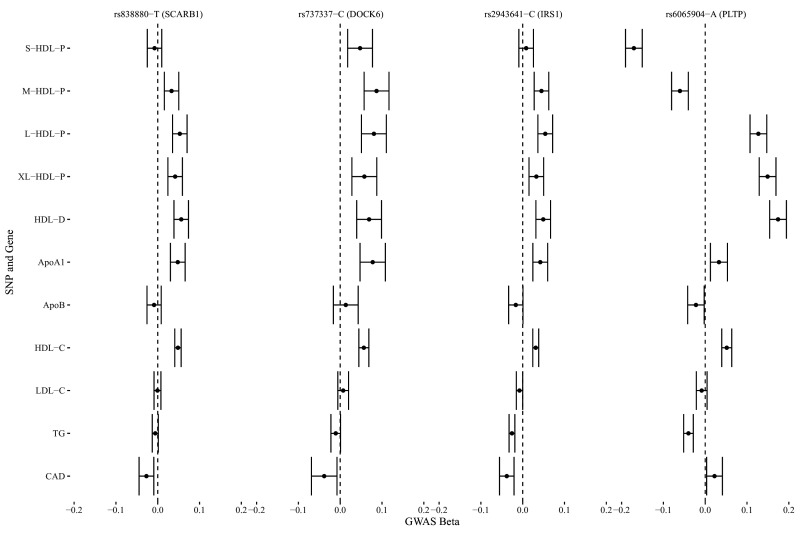
Genetic markers for HDL size (with risk alleles) and their associations with various lipid traits.

We identified four genetic variants that are associated with S-HDL-P, M-HDL-P, or HDL-D, not associated with LDL-C or ApoB, and associated with CAD: rs838880 (*SCARB1*), rs737337 (*DOCK6*), rs2943641 (*IRS1*), and rs6065904 (*PLTP*) (Figure 3). These SNP-cis gene pairs are also supported by examining expression quantitative trait loci (eQTL) in the tissue-specific GTEx data (Appendix 4). The first three variants were not associated with S-HDL-P. However, they had uniformly positive associations with M-HDL-P, L-HDL-P, XL-HDL-P, HDL-D, ApoA1, and HDL-C, and a negative association with CAD. The last variant rs6065904 had positive associations with S-HDL-P and M-HDL-P, negative associations with L-HDL-P, XL-HDL-P, HDL-D, negative but smaller associations with ApoA1 and HDL-C, and a negative association with CAD.

### Sensitivity and replicability analysis

We also investigated the effects of lipoprotein subfractions and particle sizes on MI/CAD using multiple GWAS datasets, MR designs and statistical methods. The results are provided in Appendix 3 and are generally in agreement with the primary results reported above. The diagnostic plots for S-HDL-P and M-HDL-P did not suggest evidence of violations of the instrument strength independent of direct effect (InSIDE) assumption (Bowden et al., 2015) made by RAPS and GRAPPLE (Appendix 4).

## Discussion

By using recent genetic data and MR, this study examines whether some lipoprotein subfractions and particle sizes, beyond the traditional lipid risk factors, may play a role in coronary artery disease. We find that VLDL subfractions have extremely high genetic correlations with blood triglyceride level and thus offer little extra value. We find some weak evidence that larger LDL particle size may have a small harmful effect on myocardial infarction and coronary artery disease.

Our main finding is that the size of HDL particles may play an important and previously undiscovered role. Although the concentration and lipid content of small and medium HDL particles appear to be positively correlated with HDL cholesterol and ApoA1, their genetic correlations are much smaller than 1, indicating possible independent biological pathway(s). Moreover, the MR analyses suggested that the small and medium HDL particles may have protective effects on CAD. We also find that larger HDL mean particle diameter may have a harmful effect on CAD. Finally, we identified four potential genetic markers for HDL particle size that are independent of LDL cholesterol and ApoB.

There has been a heated debate on the role of HDL particles in CAD in recent years following the failure of several trials for *CETP* inhibitors (Barter et al., 2007; Schwartz et al., 2012; Lincoff et al., 2017) and recombinant ApoA1 (Nicholls et al., 2018) targeting HDL cholesterol. Observational epidemiology studies have long demonstrated strong inverse association between HDL cholesterol and the risk of CAD or MI (Miller and Miller, 1975; Lewington et al., 2007; Di Angelantonio et al., 2009), but conflicting evidence has been found in MR studies. In an influential study, Voight and collaborators found that the genetic variants associated with HDL cholesterol had varied associations with CAD and that almost all variants suggesting a protective effect of HDL cholesterol were also associated with LDL cholesterol or triglycerides (Voight et al., 2012). Other MR studies also found that the effect of HDL cholesterol on CAD is heterogeneous (Zhao et al., 2019b) or attenuated after adjusting for LDL cholesterol and triglycerides (Holmes et al., 2017; White et al., 2016).

Notice that the harmful effect of larger HDL particle diameter found in this study relies on including HDL-C or ApoA1 in the multivariable MR analysis. Thus, the role of HDL particles in preventing CAD may be more complicated than, for example, that of LDL cholesterol or ApoB. It is possible that HDL cholesterol, HDL subfractions, and HDL particle size are all phenotypic markers for some underlying causal mechanism. A related theory is the HDL function hypothesis (Rader and Hovingh, 2014). Cholesterol efflux capacity, a measure of HDL function, has been documented as superior to HDL-C in predicting CVD risk (Rohatgi et al., 2014; Saleheen et al., 2015). Recent epidemiologic studies found that HDL particle size is positively associated with cholesterol efflux capacity in post-menopausal women (El Khoudary et al., 2016) and in an asymptomatic older cohort (Mutharasan et al., 2017). However, mechanistic efflux studies showed that small HDL particles actually mediate more cholesterol efflux (Favari et al., 2009; Du et al., 2015). A likely explanation of this seeming contradiction is that a high concentration of small HDL particles in the serum may mark a block in maturation of small HDL particles (Mutharasan et al., 2017). This can also partly explain our finding that small HDL traits have a smaller effect than medium HDL traits, as increased medium HDL might indicate successful maturation of small HDL particles.

Among the reported genetic markers, *SCARB1* and *PLTP* have established relations to HDL metabolism and CAD. *SCARB1* encodes a plasma membrane receptor for HDL and is involved in hepatic uptake of cholesterol from peripheral tissues. Recently, a rare mutation (P376L) of *SCARB1* was reported to raise HDL-C level and increase CAD risk (Zanoni et al., 2016; Samadi et al., 2019). This is opposite direction to the conventional belief that HDL-C is protective and could be explained by HDL dysfunction. *PLTP* encodes the phospholipid transfer protein and mediates the transfer of phospholipid and cholesterol from LDL and VLDL to HDL. As a result, *PLTP* plays a complex but pivotal role in HDL particle size and composition. Several studies have suggested that high *PLTP* activity is a risk factor for CAD (Schlitt et al., 2003; Schlitt et al., 2009; Zhao et al., 2019a).

Our study should be viewed in the context of its limitations, in particular, the inherent limitations of the summary-data MR design. Any causal inference from non-experimental data makes unverifiable assumptions, so does our study. Conventional MR studies assume that the genetic variants are valid instrumental variables. The statistical methods used by us make less stringent assumptions about the instrumental variables, but those assumptions could still be violated even though our model diagnosis does not suggest evidence against the InSIDE assumption. Our study did not adjust for other risk factors for CAD such as body mass index, blood pressure, and smoking. All the GWAS datasets used in this study are from the European population, so the same conclusions might not generalize to other populations. Furthermore, our study used GWAS datasets from heterogeneous subpopulations, which may also introduce bias (Zhao et al., 2019c). We also did not use more than one subfraction traits as exposures in multivariable MR because of their high genetic correlations. Alternative statistical methods could be used to select the best causal risk factor from high-throughput experiments (Zuber et al., 2019). Finally, as pointed out by revieweres, triglycerides has a greater intra-individual biological variability than HDL particle size. It is likely that triglycerides and HDL size represent a gene/environment interaction with a very large environmental component. Further investigations are needed to fully understand this mechanism.

Recently, a NMR spectroscopy method has been developed to estimate HDL cholesterol efflux capacity from serum (Kuusisto et al., 2019). That method can form the basis of a genetic analysis of HDL cholesterol efflux capacity and may complement the results here. We believe more laboratorial and epidemiological research is needed to clarify the roles of HDL subfractions and particle size in cardiovascular diseases.

## Figures and Tables

**Appendix 2—figure 1.**
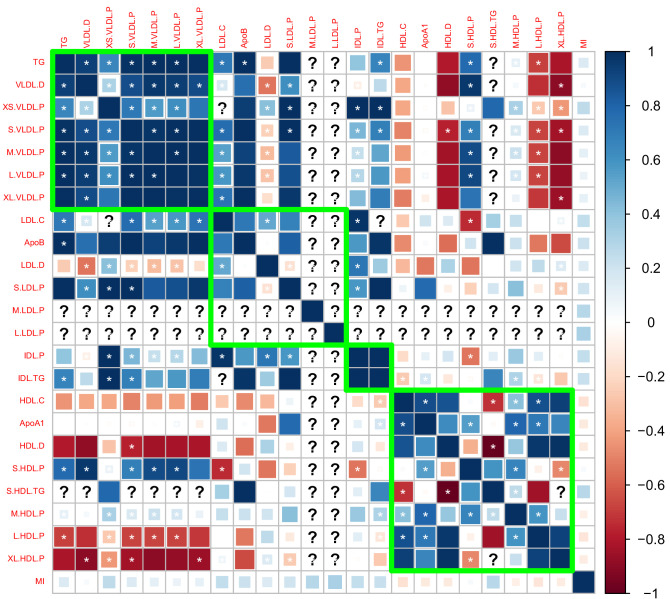
Genetic correlations computed using the Davis et al., 2017 GWAS summary dataset.

**Appendix 2—figure 2.**
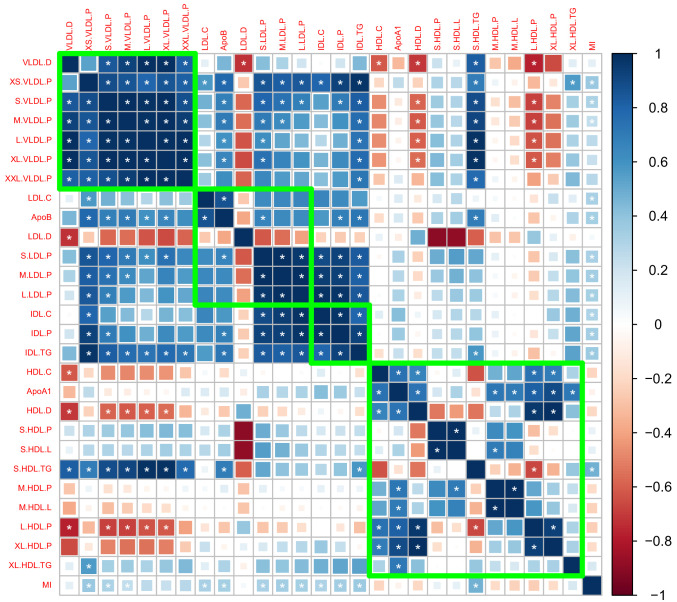
Genetic correlations computed using the Kettunen et al., 2016 GWAS summary dataset.

**Appendix 2—figure 3.**
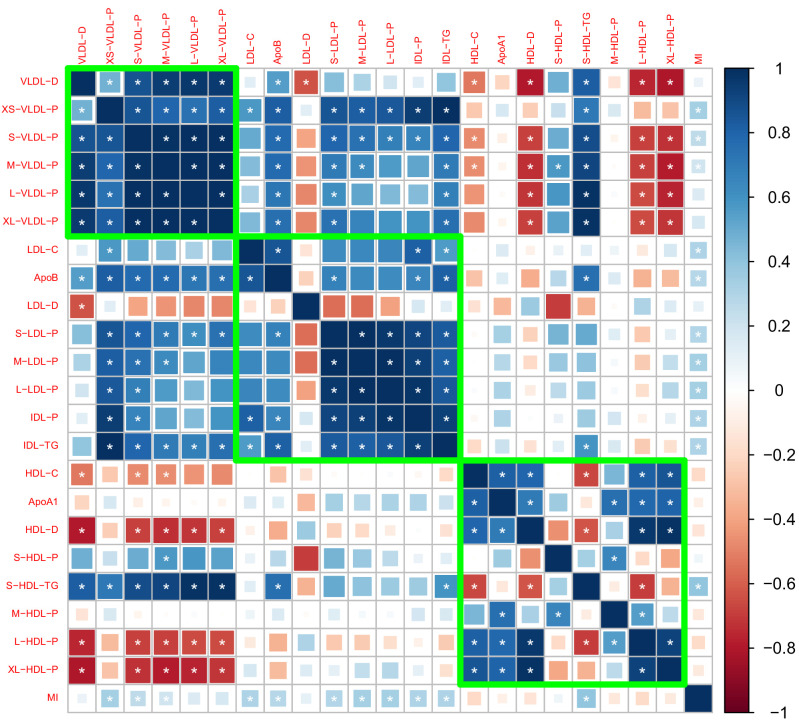
Genetic correlations computed by meta-analyzing the results in Appendix 2—figures 1 and 2.

**Appendix 3—figure 1.**
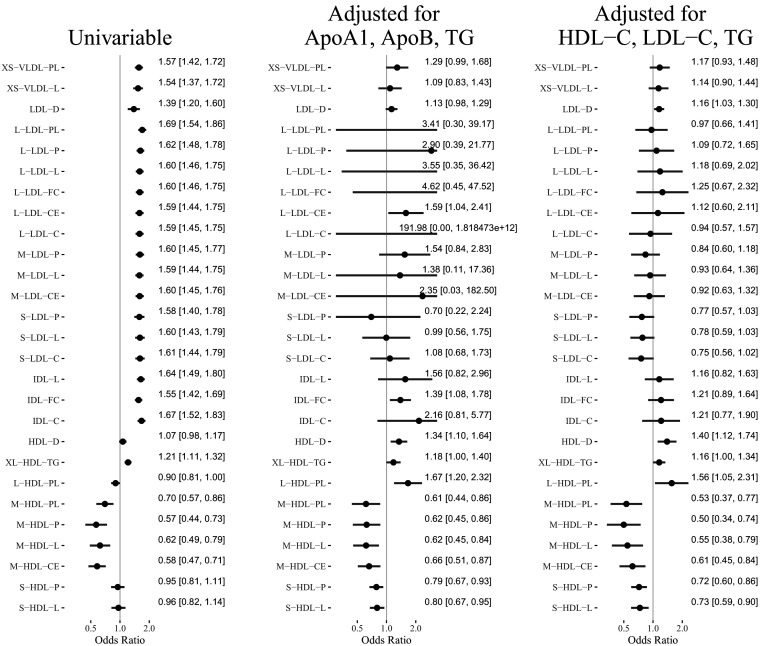
Mendelian randomization results for the 27 lipoprotein measurements selected in phenotypic screening.

**Appendix 4—figure 1.**
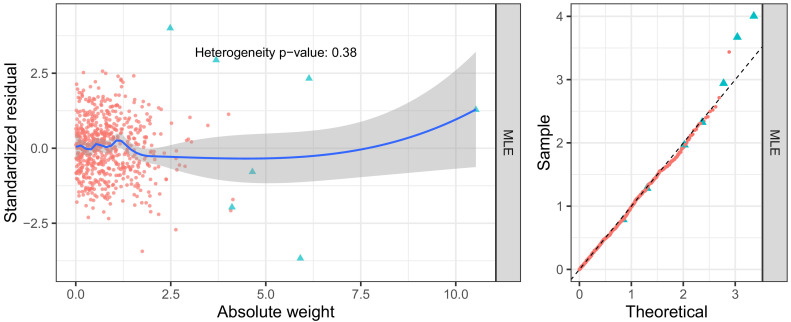
Diagnostic plots for S-HDL-P (selection: Davis; exposure: Kettunen; outcome: UK Biobank).

**Appendix 4—figure 2.**
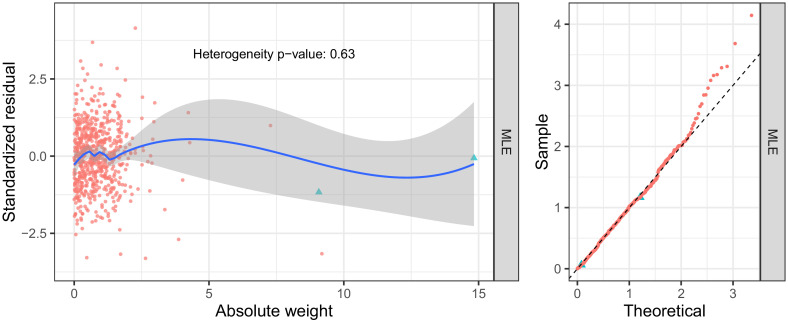
Diagnostic plots for M-HDL-P (selection: Davis; exposure: Kettunen; outcome: UK Biobank).

**Appendix 4—figure 3.**
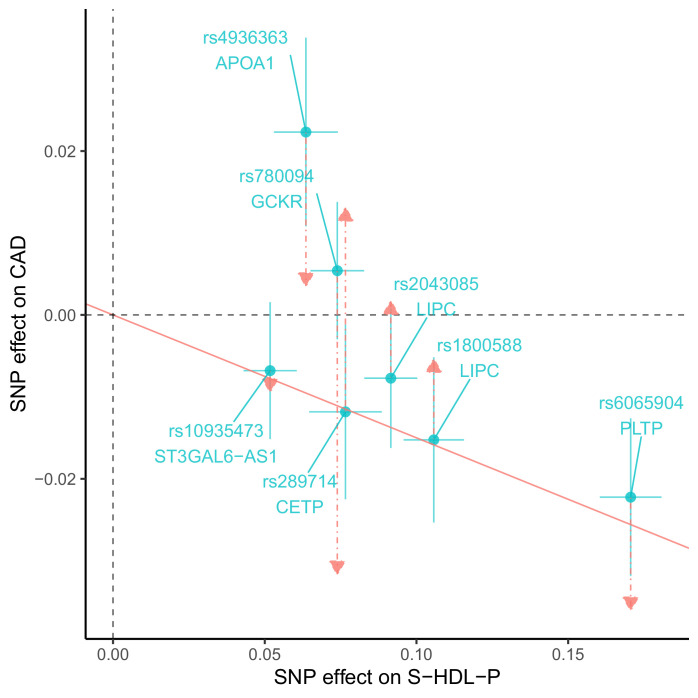
Scatter-plots for S-HDL-P with the effects on CAD adjusted for LDL-C and TG. Red lines correspond the fitted effects of S-HDL-P in multivariable MR.

**Appendix 4—figure 4.**
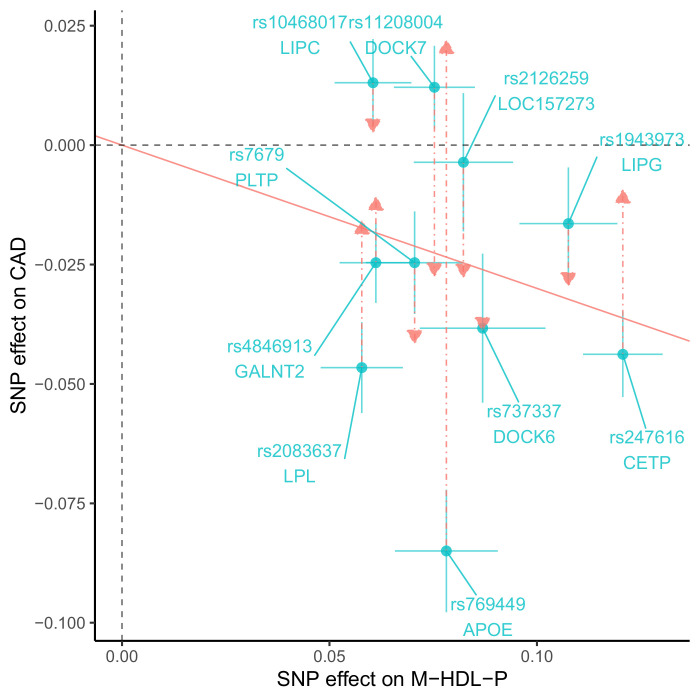
Scatter-plots for M-HDL-P with the effects on CAD adjusted for LDL-C and TG. Red lines correspond the fitted effects of M-HDL-P in multivariable MR.

## Data Availability

GWAS data used in the data are publicly available. Details can be found in Table 1.

## Additional files

Transparent reporting form

## Appendix 1

Lipid and lipoprotein traits Two published GWAS of the human lipidome [Kettunen2016, Davis2017] measured lipoprotein subfractions and particle sizes using NMR spectroscopy. We investigated the 82 lipid and lipoprotein traits measured in these studies that are related to very-low-density lipoprotein (VLDL), LDL, and HDL subfractions and particle sizes. All the subfraction traits are named using three components separated by hyphen: the first indicates the size (XS, S, M, L, XL, XXL); the second indicates the category according to the lipoprotein density (VLDL, LDL, IDL, HDL); the third indicates the measurement (C for total cholesterol, CE for cholesterol esters, FC for free cholesterol, L for total lipids, P for particle concentration, PL for phospholipids, TG for triglycerides). A full list of lipid and lipoprotein traits used in our study can be found in Appendix 1—table 1 below.

**Appendix 1—table 1.** All 82 traits included in this study and whether they are measured in the Kettunen and Davis GWAS (NA means not available).

Trait	Description	Kettunen	Davis
VLDL traits and total triglycerides			
TG	Total triglycerides		
VLDL-D	VLDL diameter		
XS-VLDL-L	Total lipids in very small VLDL		NA
XS-VLDL-P	Concentration of very small VLDL particles		
XS-VLDL-PL	Phospholipids in very small VLDL		
XS-VLDL-TG	Triglycerides in very small VLDL		
S-VLDL-C	Total cholesterol in small VLDL		
S-VLDL-FC	Free cholesterol in small VLDL		
S-VLDL-L	Total lipids in small VLDL		NA
S-VLDL-P	Concentration of small VLDL particles		
S-VLDL-PL	Phospholipids in small VLDL		
S-VLDL-TG	Triglycerides in small VLDL		
M-VLDL-C	Total cholesterol in medium VLDL		
M-VLDL-CE	Cholesterol esters in medium VLDL		
M-VLDL-FC	Free cholesterol in medium VLDL		
M-VLDL-L	Total lipids in medium VLDL		NA
M-VLDL-P	Concentration of medium VLDL particles		
M-VLDL-PL	Phospholipids in medium VLDL		
M-VLDL-TG	Triglycerides in medium VLDL		
L-VLDL-C	Total cholesterol in large VLDL		
L-VLDL-CE	Cholesterol esters in large VLDL		
L-VLDL-FC	Free cholesterol in large VLDL		
L-VLDL-L	Total lipids in large VLDL		NA
L-VLDL-P	Concentration of large VLDL particles		
L-VLDL-PL	Phospholipids in large VLDL		
L-VLDL-TG	Triglycerides in large VLDL		
XL-VLDL-L	Total lipids in very large VLDL		NA
XL-VLDL-P	Concentration of very large VLDL particles		
XL-VLDL-PL	Phospholipids in very large VLDL		
XL-VLDL-TG	Triglycerides in very large VLDL		
XXL-VLDL-L	Total lipids in chylomicrons and extremely very large VLDL		NA
XXL-VLDL-P	Concentration of chylomicrons and extremely very large VLDL particles		
XXL-VLDL-PL	Phospholipids in chylomicrons and extremely very large		
XXL-VLDL-TG	Triglycerides in chylomicrons and extremely very large		
LDL and IDL traits			
LDL-C	Total cholesterol in LDL		
ApoB	Apolipoprotein B		
LDL-D	LDL diameter		
S-LDL-C	Total cholesterol in small LDL		
S-LDL-L	Total lipids in small LDL		NA
S-LDL-P	Phospholipids in small LDL		
M-LDL-C	Total cholesterol in medium LDL		
M-LDL-CE	Cholesterol esters in medium LDL		
M-LDL-L	Total lipids in medium LDL		NA
M-LDL-P	Concentration of medium LDL particles		
M-LDL-PL	Phospholipids in medium LDL		
L-LDL-C	Total cholesterol in large LDL		
L-LDL-CE	Cholesterol esters in large LDL		
L-LDL-FC	Free cholesterol in large LDL		
L-LDL-L	Total lipids in large LDL		NA
L-LDL-P	Concentration of large LDL particles		
L-LDL-PL	Phospholipids in large LDL		
IDL-C	Total cholesterol in IDL		
IDL-FC	Free cholesterol in IDL		
IDL-L	Total lipids in IDL		NA
IDL-P	Concentration of IDL particles		
IDL-PL	Phospholipids in IDL		
IDL-TG	Triglycerides in IDL		
HDL traits			
HDL-C	Total cholesterol in HDL		
ApoA1	Apolipoprotein A1		
HDL-D	HDL diameter		
S-HDL-L	Total lipids in small HDL		NA
S-HDL-P	Concentration of small HDL particles		
S-HDL-TG	Triglycerides in small HDL		
M-HDL-C	Total cholesterol in medium HDL		
M-HDL-CE	Cholesterol esters in medium HDL		
M-HDL-FC	Free cholesterol in medium HDL		
M-HDL-L	Total lipids in medium HDL		NA
M-HDL-P	Concentration of medium HDL particles		
M-HDL-PL	Phospholipids in medium HDL		
L-HDL-C	Total cholesterol in large HDL		
L-HDL-CE	Cholesterol esters in large HDL		
L-HDL-FC	Free cholesterol in large HDL		
L-HDL-L	Total lipids in large HDL		NA
L-HDL-P	Concentration of large HDL particles		
L-HDL-PL	Phospholipids in large HDL		
XL-HDL-C	Total cholesterol in very large HDL		
XL-HDL-CE	Cholesterol esters in very large HDL		
XL-HDL-FC	Free cholesterol in very large HDL		
XL-HDL-L	Total lipids in very large HDL		NA
XL-HDL-P	Concentration of very large HDL particles		
XL-HDL-PL	Phospholipids in very large HDL		
XL-HDL-TG	Triglycerides in very large HDL		

## Appendix 2

Genetic correlations We estimated the genetic correlation between lipoprotein subfractions, particle sizes, and traditional lipid risk factors using the LD score regression (Li et al., 2016). Appendix 2—figure 1–3 show the estimated genetic correlation matrix between selected traits using different datasets. Below the figures, Appendix 2—table 1 shows the estimated genetic correlations of the lipoprotein subbfractions with the traditional lipid risk factors using the Davis GWAS. The results in Appendix 2—table 1 were then used to screen the traits as described in Materials and methods.

**Appendix 2—table 1.** Estimated genetic correlation (standard error) of the lipoprotein subfractions with the traditional lipid risk factors using the Davis GWAS. Bolded estimates are above 0.8 and the corresponding traits were removed in phenotypic screening.

Trait	ApoA1	ApoB	HDL-C	LDL-C	TG
S-HDL-L	0.31 (0.28)	0.34 (0.25)	0.13 (0.26)	0.27 (0.3)	0.2 (0.22)
S-HDL-P	0.36 (0.24)	0.27 (0.22)	−0.01 (0.22)	0.1 (0.31)	0.48 (0.17)
S-HDL-TG	−0.13 (0.25)	0.77 (0.13)	−0.66 (0.15)	0.13 (0.28)	1.03 (0.07)
M-HDL-C	0.65 (0.14)	−0.18 (0.2)	0.81 (0.09)	−0.09 (0.25)	−0.34 (0.17)
M-HDL-CE	0.68 (0.14)	−0.23 (0.21)	0.57 (0.12)	−0.24 (0.24)	−0.32 (0.18)
M-HDL-FC	0.67 (0.12)	−0.08 (0.21)	0.83 (0.08)	0.04 (0.24)	−0.28 (0.18)
M-HDL-L	0.71 (0.15)	0.02 (0.27)	0.52 (0.17)	−0.03 (0.29)	−0.19 (0.25)
M-HDL-P	0.75 (0.12)	0.15 (0.23)	0.46 (0.14)	0.08 (0.26)	0 (0.19)
M-HDL-PL	0.69 (0.13)	0.04 (0.22)	0.65 (0.11)	0.02 (0.25)	−0.04 (0.19)
L-HDL-C	0.76 (0.11)	−0.42 (0.13)	0.95 (0.02)	−0.1 (0.18)	−0.62 (0.09)
L-HDL-CE	0.82 (0.1)	−0.4 (0.12)	0.93 (0.04)	−0.16 (0.17)	−0.62 (0.09)
L-HDL-FC	0.66 (0.12)	−0.46 (0.13)	0.92 (0.03)	−0.13 (0.18)	−0.7 (0.08)
L-HDL-L	0.81 (0.11)	−0.29 (0.15)	0.74 (0.07)	−0.15 (0.18)	−0.56 (0.12)
L-HDL-P	0.79 (0.09)	−0.35 (0.13)	0.82 (0.05)	−0.12 (0.16)	−0.61 (0.09)
L-HDL-PL	0.77 (0.09)	−0.34 (0.13)	0.79 (0.05)	−0.12 (0.17)	−0.61 (0.09)
XL-HDL-C	0.75 (0.16)	−0.25 (0.19)	0.9 (0.1)	0.4 (0.27)	−0.63 (0.13)
XL-HDL-CE	0.82 (0.16)	−0.17 (0.19)	0.82 (0.09)	0.41 (0.27)	−0.54 (0.12)
XL-HDL-FC	0.72 (0.14)	−0.37 (0.18)	0.94 (0.08)	0.17 (0.23)	−0.71 (0.11)
XL-HDL-L	0.93 (0.16)	−0.08 (0.25)	0.68 (0.14)	0.1 (0.27)	−0.35 (0.2)
XL-HDL-P	0.81 (0.13)	−0.32 (0.16)	0.86 (0.08)	0.17 (0.21)	−0.69 (0.11)
XL-HDL-PL	0.76 (0.12)	−0.41 (0.15)	0.83 (0.07)	−0.09 (0.18)	−0.7 (0.09)
XL-HDL-TG	0.72 (0.13)	0.49 (0.17)	0.33 (0.13)	0.13 (0.26)	0.3 (0.15)
HDL-D	0.7 (0.11)	−0.36 (0.13)	0.8 (0.06)	−0.08 (0.17)	−0.64 (0.09)
IDL-C	0.38 (0.21)	0.58 (0.19)	0.07 (0.19)	0.8 (0.14)	0.39 (0.17)
IDL-FC	0.23 (0.2)	0.78 (0.12)	−0.05 (0.17)	0.61 (0.19)	0.44 (0.15)
IDL-L	0.38 (0.23)	0.65 (0.18)	0.05 (0.2)	0.64 (0.2)	0.47 (0.17)
IDL-P	0.31 (0.2)	0.66 (0.14)	−0.04 (0.17)	0.82 (0.13)	0.49 (0.14)
IDL-PL	0.25 (0.23)	0.83 (0.1)	−0.12 (0.19)	0.7 (0.19)	0.64 (0.15)
IDL-TG	0.22 (0.18)	0.82 (0.08)	−0.2 (0.13)	0.56 (0.15)	0.67 (0.08)
S-LDL-C	0.11 (0.28)	0.66 (0.18)	−0.16 (0.22)	0.44 (0.34)	0.58 (0.14)
S-LDL-L	0.26 (0.23)	0.66 (0.17)	−0.06 (0.21)	0.62 (0.21)	0.58 (0.13)
S-LDL-P	0.34 (0.2)	0.68 (0.15)	−0.02 (0.19)	0.63 (0.18)	0.58 (0.13)
M-LDL-C	0.15 (0.26)	0.63 (0.18)	0.22 (0.22)	0.87 (0.08)	0.13 (0.23)
M-LDL-CE	0.3 (0.23)	0.61 (0.2)	0.05 (0.21)	0.65 (0.2)	0.45 (0.16)
M-LDL-L	0.29 (0.22)	0.63 (0.18)	0.01 (0.21)	0.66 (0.19)	0.5 (0.15)
M-LDL-P	0.29 (0.23)	0.63 (0.18)	−0.01 (0.21)	0.65 (0.21)	0.51 (0.15)
M-LDL-PL	0.2 (0.24)	0.69 (0.16)	0.11 (0.2)	0.89 (0.06)	0.18 (0.22)
L-LDL-C	0.25 (0.24)	0.58 (0.21)	0.25 (0.22)	0.68 (0.19)	0.23 (0.21)
L-LDL-CE	0.3 (0.23)	0.58 (0.22)	0.05 (0.21)	0.65 (0.21)	0.41 (0.17)
L-LDL-FC	0.31 (0.24)	0.57 (0.22)	0.33 (0.23)	0.7 (0.18)	0.13 (0.23)
L-LDL-L	0.31 (0.23)	0.61 (0.2)	0.04 (0.21)	0.65 (0.21)	0.44 (0.17)
L-LDL-P	0.31 (0.23)	0.63 (0.19)	0.02 (0.21)	0.65 (0.21)	0.47 (0.16)
L-LDL-PL	0.27 (0.25)	0.61 (0.2)	0.24 (0.22)	0.67 (0.2)	0.27 (0.2)
LDL-D	−0.33 (0.25)	−0.22 (0.23)	−0.15 (0.21)	−0.15 (0.29)	−0.37 (0.16)
XS-VLDL-L	0.25 (0.23)	0.8 (0.08)	−0.2 (0.17)	0.61 (0.14)	0.73 (0.09)
XS-VLDL-P	0.17 (0.18)	0.83 (0.07)	−0.26 (0.13)	0.57 (0.13)	0.71 (0.07)
XS-VLDL-PL	0.21 (0.19)	0.78 (0.09)	−0.15 (0.15)	0.74 (0.14)	0.57 (0.11)
XS-VLDL-TG	0.06 (0.18)	0.83 (0.08)	−0.37 (0.11)	0.56 (0.13)	0.85 (0.04)
S-VLDL-FC	−0.08 (0.2)	0.94 (0.05)	−0.49 (0.12)	0.59 (0.12)	0.92 (0.03)
S-VLDL-L	−0.12 (0.24)	0.7 (0.08)	−0.46 (0.15)	0.5 (0.14)	0.8 (0.05)
S-VLDL-P	−0.09 (0.19)	0.78 (0.07)	−0.48 (0.11)	0.5 (0.14)	0.95 (0.02)
S-VLDL-PL	−0.03 (0.2)	0.82 (0.08)	−0.43 (0.12)	0.44 (0.17)	0.92 (0.03)
S-VLDL-TG	−0.1 (0.2)	0.9 (0.08)	−0.49 (0.11)	0.49 (0.15)	0.98 (0.01)
S-VLDL-C	0.01 (0.2)	0.9 (0.06)	−0.39 (0.13)	0.61 (0.15)	0.89 (0.05)
M-VLDL-C	−0.01 (0.2)	0.8 (0.09)	−0.47 (0.12)	0.41 (0.18)	0.95 (0.02)
M-VLDL-CE	0.01 (0.19)	0.78 (0.08)	−0.43 (0.12)	0.5 (0.15)	0.9 (0.03)
M-VLDL-FC	0 (0.21)	0.83 (0.09)	−0.48 (0.12)	0.4 (0.18)	0.97 (0.01)
M-VLDL-L	−0.1 (0.24)	0.66 (0.11)	−0.48 (0.15)	0.4 (0.18)	0.8 (0.05)
M-VLDL-P	−0.06 (0.19)	0.78 (0.1)	−0.46 (0.12)	0.43 (0.16)	0.98 (0.02)
M-VLDL-PL	0.03 (0.21)	0.85 (0.09)	−0.48 (0.12)	0.4 (0.18)	0.98 (0.01)
M-VLDL-TG	−0.02 (0.21)	0.82 (0.11)	−0.5 (0.13)	0.33 (0.19)	0.98 (0.02)
L-VLDL-C	−0.05 (0.2)	0.83 (0.12)	−0.55 (0.12)	0.36 (0.19)	1 (0.02)
L-VLDL-CE	0 (0.19)	0.78 (0.12)	−0.44 (0.12)	0.43 (0.19)	0.93 (0.03)
L-VLDL-FC	−0.03 (0.2)	0.84 (0.12)	−0.53 (0.13)	0.36 (0.19)	1 (0.02)
L-VLDL-L	−0.06 (0.24)	0.66 (0.14)	−0.47 (0.16)	0.36 (0.2)	0.86 (0.05)
L-VLDL-P	−0.02 (0.21)	0.72 (0.12)	−0.44 (0.13)	0.33 (0.18)	0.98 (0.02)
L-VLDL-PL	0.01 (0.21)	0.86 (0.12)	−0.53 (0.13)	0.3 (0.2)	1.04 (0.03)
L-VLDL-TG	−0.06 (0.21)	0.78 (0.12)	−0.54 (0.13)	0.26 (0.19)	1 (0.02)
XL-VLDL-L	−0.08 (0.24)	0.7 (0.15)	−0.52 (0.16)	0.43 (0.2)	0.85 (0.05)
XL-VLDL-P	−0.06 (0.2)	0.76 (0.12)	−0.48 (0.13)	0.44 (0.18)	0.95 (0.03)
XL-VLDL-PL	−0.09 (0.23)	0.82 (0.13)	−0.62 (0.15)	0.32 (0.21)	1.06 (0.04)
XL-VLDL-TG	−0.14 (0.21)	0.86 (0.13)	−0.65 (0.13)	0.34 (0.19)	1.03 (0.04)
XXL-VLDL-L	−0.07 (0.25)	0.65 (0.16)	−0.5 (0.17)	0.38 (0.22)	0.83 (0.06)
XXL-VLDL-P	0.17 (0.2)	0.72 (0.15)	−0.3 (0.15)	0.39 (0.21)	0.86 (0.07)
XXL-VLDL-PL	−0.3 (0.24)	0.66 (0.17)	−0.8 (0.16)	0.22 (0.21)	1.06 (0.06)
XXL-VLDL-TG	−0.21 (0.25)	0.64 (0.16)	−0.7 (0.15)	0.22 (0.22)	1.08 (0.05)
VLDL-D	−0.22 (0.2)	0.55 (0.14)	−0.53 (0.12)	0.12 (0.19)	0.86 (0.04)

## Appendix 3

Mendelian randomization We implemented several Mendelian randomization (MR) designs and statistical methods to estimate the causal effect of lipoprotein subfractions and particles sizes on coronary artery disease. In general, we adopted the three-sample summary data MR design described in Zhao et al., 2019b, Wang et al., 2020 and we swapped the roles of the GWAS datasets whenever permitted by the statistical methods. More specifically, the statistical methods we used for univariable MR (RAPS, IVW, weighted median) require that the GWAS datasets for obtaining instruments, SNP effects on the exposure, and SNP effects on the outcome must have no overlapping sample. The multivariable MR method we used (GRAPPLE) allows the exposure and outcome GWAS to be dependent and estimates the proportion of overlapping sample. However, GRAPPLE still requires that the selection GWAS uses an non-overlapping sample. The MR designs we implemented in this study are summarized in Appendix 3—table 1. We considered two ways of instrument selection for univariable MR. In ‘traditional selection’, the traditional lipid traits were used to select the instruments for the corresponding subfraction traits. That is, HDL-C was used to select SNPs for HDL subfractions and particle size, LDL-C for IDL and LDL subfractions and particle size, and TG for VLDL subfractions and particle size. This tends to select more instruments because the GWAS for traditional lipid traits had a larger sample size. In ‘subfraction selection’, the instrumental SNPs were selected for each lipoprotein subfraction and particle size using the same or closest trait in the selection GWAS. For example, if the exposure under investigation is S-HDL-L but it is not measured in the Davis GWAS (if it is used for selection), S-HDL-P is used instead for instrument selection. For multivariable MR, we considered two models with different sets of exposures: TG, LDL-C, HDL-C, and the subfraction/particle size under investigation; TG, ApoB, ApoA1, and the subfraction/particle size under investigation. SNPs were selected as potential instruments if they were associated (p-value $\leq 10^{-4}$) with at least one of the four exposures. LD clumping was then used to obtain independent instruments, as described in Materials and Methods. We briefly comment on the statistical methods used in univariable MR. All the three methods we used—RAPS, IVW, weighted median—require that the exposure GWAS and outcome GWAS have non-overlapping samples. RAPS and weighted median can provide consistent estimate of the causal effect even when some of the genetic variants are not valid instruments, provided that the direct effects of the genetic variants are independent of the strength of their associations with the exposure. The last condition is called the Instrument Strength Independent of Direct Effect (InSIDE) assumption in the MR literature [bowden2015mendelian]. RAPS is also robust to idiosyncratically large direct effect (Bowden et al., 2015). Because IVW and weighted median can be severely biased by weak instruments (Zhao et al., 2020), we only used them with the set of SNPs that have genome-wide significant association (p-value $\leq 5\times 10^{-8}$) with the exposure. In comparison, RAPS does not suffer from weak instrument bias and we used it with all the SNPs obtained by LD clumping without any p-value threshold. Below, Appendix 3—figure 1 shows the MR results for the 27 lipoprotein measurements selected in phenotypic screening. Estimates that are statistically significant at a false discovery rate of 0.05 are shown in Figure 2 of the main paper. Appendix 3—table 2 shows the estimated effect of all the lipoprotein subfractions and particle sizes on myocardial infarction or coronary artery disease in various MR designs. Full results of the multivariable MR analyses, including the estimated effects of the traditional lipid risk factors, can be found in Appendix 3—tables 5 and 6. The results of the univariable MR analyses using IVW and weighted median estimators can be found in Appendix 3—tables 3 and 4.

**Appendix 3—table 1.** Three-sample Mendelian randomization designs.

MR design	Selection	Exposure	Outcome	Reported in
Univariable(traditional selection)	GERA	Davis	CARDIoGRAMplusC4D	Appendix 3—table 2–4
	GERA	Davis	UK Biobank	Appendix 3—table 2–4
	GERA	Kettunen	UK Biobank	Appendix 3—table 2–4
	GLGC	Davis	UK Biobank	Appendix 3—table 2–4
Univariable(subfraction selection)	Davis	Kettunen	UK Biobank	Figure 2; Appendix 3—figure 1 and Appendix 3—table 2–4
	Kettunen	Davis	UK Biobank	Appendix 3—figure 1 and Appendix 3—table 2–4
Multivariable	Davis, GERA	Kettunen, GLGC	CARDIoGRAMplusC4D+ UK Biobank	Figure 2, Table 2; Appendix 3—figure 1 and Appendix 3—table 2–4 Pooled results In the tables below, Red indicates p-value is significant (at level 0.05) after Bonferroni correction for all the results in the corresponding table and blue indicates p-value ≤ 0.05.

**Appendix 3—table 2.** Mendelian randomization results using all selected SNPs (univariable MR using RAPS and multivariable MR using GRAPPLE).

	Method: RAPS/GRAPPLE + All SNPs							
Screening	GERA	GERA	GERA	GLGC	Davis	Kettunen	GERA + Davis	GERA + Davis
Exposure	Davis	Davis	Kettunen	Davis	Kettunen	Davis	GLGC + Kettunen	GLGC + Kettunen
Outcome	CAD	UKB	UKB	UKB	UKB	UKB	CAD + UKB	CAD + UKB
Adjusted							HDL-C + LDL-C + TG	ApoA1 + ApoB + TG
VLDL traits								
TG	0.258 (0.053)	0.296 (0.075)	NA	0.262 (0.06)	NA	0.289 (0.068)	NA	NA
VLDL-D	-0.099 (0.049)	0.028 (0.074)	0.072 (0.073)	0.116 (0.065)	-0.163 (0.067)	-0.204 (0.071)	-0.588 (0.094)	-0.32 (0.112)
XS-VLDL-L	NA	NA	0.368 (0.064)	NA	0.429 (0.059)	NA	0.132 (0.119)	0.084 (0.141)
XS-VLDL-P	0.17 (0.031)	0.26 (0.048)	0.367 (0.065)	0.248 (0.047)	0.429 (0.06)	0.338 (0.056)	0.118 (0.125)	0.061 (0.158)
XS-VLDL-PL	0.191 (0.034)	0.284 (0.055)	0.386 (0.069)	0.278 (0.052)	0.449 (0.049)	0.435 (0.049)	0.159 (0.12)	0.253 (0.135)
XS-VLDL-TG	0.201 (0.034)	0.3 (0.053)	0.388 (0.068)	0.283 (0.046)	0.372 (0.063)	0.326 (0.055)	-0.157 (0.187)	-0.248 (0.15)
S-VLDL-C	0.294 (0.06)	0.343 (0.076)	NA	0.322 (0.063)	NA	0.424 (0.094)	-1.035 (0.323)	-1.265 (0.568)
S-VLDL-FC	0.243 (0.051)	0.303 (0.068)	0.389 (0.079)	0.286 (0.056)	0.489 (0.071)	0.416 (0.074)	-1.027 (0.337)	-0.489 (0.213)
S-VLDL-L	NA	NA	0.356 (0.075)	NA	0.376 (0.072)	NA	-0.898 (0.28)	-1.629 (0.586)
S-VLDL-P	0.226 (0.047)	0.288 (0.068)	0.343 (0.074)	0.261 (0.054)	0.359 (0.069)	0.271 (0.094)	-1.245 (0.463)	-1.644 (0.606)
S-VLDL-PL	0.228 (0.047)	0.294 (0.067)	0.372 (0.074)	0.273 (0.054)	0.365 (0.066)	0.336 (0.063)	-0.613 (0.182)	-1.213 (0.478)
S-VLDL-TG	0.223 (0.049)	0.283 (0.071)	0.323 (0.073)	0.25 (0.055)	0.327 (0.071)	0.275 (0.067)	NaN	-0.301 (0.108)
M-VLDL-C	0.253 (0.053)	0.304 (0.078)	0.327 (0.074)	0.276 (0.06)	0.368 (0.07)	0.312 (0.079)	-1.433 (0.451)	-0.373 (0.118)
M-VLDL-CE	0.248 (0.051)	0.309 (0.074)	0.344 (0.077)	0.285 (0.058)	0.369 (0.073)	0.295 (0.069)	-1.035 (0.293)	-0.995 (0.338)
M-VLDL-FC	0.245 (0.058)	0.283 (0.082)	0.31 (0.076)	0.259 (0.063)	0.341 (0.069)	0.341 (0.068)	-1.412 (0.444)	-0.799 (0.311)
M-VLDL-L	NA	NA	0.311 (0.079)	NA	0.358 (0.078)	NA	-1.878 (0.75)	-0.298 (0.098)
M-VLDL-P	0.25 (0.062)	0.282 (0.083)	0.305 (0.081)	0.247 (0.065)	0.293 (0.089)	0.269 (0.065)	-1.974 (0.745)	-0.312 (0.096)
M-VLDL-PL	0.248 (0.056)	0.295 (0.077)	0.318 (0.075)	0.259 (0.06)	0.351 (0.071)	0.31 (0.063)	-2.012 (0.943)	-0.297 (0.106)
M-VLDL-TG	0.205 (0.064)	0.248 (0.087)	0.3 (0.082)	0.224 (0.067)	0.275 (0.092)	0.246 (0.074)	-2.133 (0.879)	-0.806 (0.455)
L-VLDL-C	0.299 (0.067)	0.304 (0.1)	0.297 (0.081)	0.291 (0.077)	0.289 (0.085)	0.317 (0.077)	-1.254 (0.297)	-0.609 (0.337)
L-VLDL-CE	0.247 (0.061)	0.282 (0.088)	0.282 (0.082)	0.282 (0.072)	0.285 (0.082)	0.3 (0.112)	-1.081 (0.282)	-0.673 (0.217)
L-VLDL-FC	0.316 (0.076)	0.294 (0.108)	0.311 (0.083)	0.287 (0.081)	0.351 (0.087)	0.298 (0.078)	-1.274 (0.308)	-0.619 (0.291)
L-VLDL-L	NA	NA	0.36 (0.096)	NA	0.32 (0.102)	NA	-1.277 (0.313)	-0.532 (0.278)
L-VLDL-P	0.268 (0.073)	0.287 (0.103)	0.281 (0.085)	0.262 (0.075)	0.219 (0.086)	0.255 (0.082)	-1.357 (0.344)	-0.617 (0.229)
L-VLDL-PL	0.322 (0.071)	0.318 (0.102)	0.346 (0.089)	0.283 (0.077)	0.397 (0.101)	0.351 (0.076)	NaN	-0.287 (0.104)
L-VLDL-TG	0.243 (0.077)	0.238 (0.104)	0.332 (0.094)	0.246 (0.08)	0.26 (0.103)	0.324 (0.082)	-1.428 (0.372)	-0.252 (0.091)
XL-VLDL-L	NA	NA	0.289 (0.098)	NA	0.435 (0.14)	NA	-1.069 (0.203)	-0.577 (0.249)
XL-VLDL-P	0.27 (0.074)	0.262 (0.099)	0.281 (0.093)	0.279 (0.084)	0.404 (0.122)	0.251 (0.084)	-1.209 (0.238)	-0.373 (0.109)
XL-VLDL-PL	0.446 (0.09)	0.344 (0.13)	0.31 (0.093)	0.361 (0.118)	0.375 (0.12)	0.408 (0.102)	-1.214 (0.257)	-0.583 (0.268)
XL-VLDL-TG	0.294 (0.092)	0.229 (0.109)	0.261 (0.094)	0.284 (0.095)	0.365 (0.111)	0.319 (0.093)	-1.071 (0.205)	-0.603 (0.248)
XXL-VLDL-L	NA	NA	0.397 (0.108)	NA	0.312 (0.108)	NA	-1.355 (0.318)	-0.402 (0.144)
XXL-VLDL-P	0.308 (0.08)	0.327 (0.096)	0.378 (0.097)	0.297 (0.088)	0.32 (0.101)	0.227 (0.073)	-1.639 (0.502)	-1.089 (0.449)
XXL-VLDL-PL	0.338 (0.091)	0.346 (0.103)	0.342 (0.103)	0.351 (0.103)	0.282 (0.114)	0.317 (0.086)	-1.259 (0.262)	-0.814 (0.344)
XXL-VLDL-TG	0.384 (0.108)	0.374 (0.124)	0.348 (0.1)	0.433 (0.121)	0.304 (0.138)	0.359 (0.18)	-1.202 (0.262)	-1.075 (0.402)
IDL/LDL traits								
LDL-C	0.523 (0.043)	0.512 (0.053)	0.514 (0.042)	0.473 (0.055)	0.435 (0.048)	0.464 (0.048)	NA	0.319 (0.182)
ApoB	0.605 (0.056)	0.55 (0.062)	0.551 (0.052)	0.543 (0.069)	0.61 (0.066)	0.613 (0.06)	-0.532 (0.191)	NA
LDL-D	0.271 (0.215)	0.452 (0.299)	2.064 (0.233)	0.831 (0.684)	0.328 (0.073)	0.201 (0.055)	0.145 (0.061)	0.119 (0.071)
S-LDL-C	0.624 (0.053)	0.589 (0.061)	0.539 (0.048)	0.537 (0.067)	0.474 (0.056)	0.48 (0.05)	-0.282 (0.152)	0.08 (0.238)
S-LDL-L	NA	NA	0.561 (0.047)	NA	0.473 (0.057)	NA	-0.251 (0.145)	-0.005 (0.29)
S-LDL-P	0.621 (0.057)	0.581 (0.065)	0.56 (0.049)	0.558 (0.073)	0.459 (0.061)	0.546 (0.063)	-0.266 (0.151)	-0.362 (0.596)
M-LDL-C	0.648 (0.055)	0.607 (0.062)	0.545 (0.044)	0.545 (0.068)	0.455 (0.049)	0.557 (0.054)	-0.271 (0.162)	-0.169 (0.909)
M-LDL-CE	0.643 (0.056)	0.601 (0.062)	0.564 (0.042)	0.545 (0.069)	0.467 (0.05)	0.55 (0.055)	-0.088 (0.188)	NaN
M-LDL-L	NA	NA	0.559 (0.042)	NA	0.461 (0.049)	NA	-0.069 (0.191)	NaN
M-LDL-P	0.638 (0.056)	0.597 (0.062)	0.557 (0.043)	0.54 (0.069)	0.472 (0.051)	0.46 (0.05)	-0.179 (0.174)	0.432 (0.31)
M-LDL-PL	0.658 (0.063)	0.605 (0.067)	0.556 (0.047)	0.571 (0.077)	0.506 (0.053)	0.559 (0.057)	-0.407 (0.162)	-0.566 (0.839)
L-LDL-C	0.627 (0.053)	0.577 (0.059)	0.515 (0.042)	0.504 (0.063)	0.465 (0.048)	0.488 (0.052)	-0.059 (0.261)	NaN
L-LDL-CE	0.638 (0.055)	0.589 (0.06)	0.555 (0.041)	0.514 (0.065)	0.463 (0.049)	0.493 (0.054)	0.116 (0.321)	0.461 (0.213)
L-LDL-FC	0.609 (0.051)	0.557 (0.057)	0.503 (0.041)	0.491 (0.06)	0.468 (0.047)	0.457 (0.052)	0.223 (0.315)	NaN
L-LDL-L	NA	NA	0.543 (0.04)	NA	0.468 (0.047)	NA	0.167 (0.273)	NaN
L-LDL-P	0.606 (0.052)	0.559 (0.058)	0.545 (0.041)	0.49 (0.062)	0.484 (0.046)	0.494 (0.048)	0.084 (0.213)	NaN
L-LDL-PL	0.61 (0.053)	0.558 (0.058)	0.515 (0.043)	0.492 (0.063)	0.528 (0.048)	0.502 (0.052)	-0.036 (0.195)	NaN
IDL-C	0.596 (0.054)	0.55 (0.059)	0.562 (0.042)	0.481 (0.064)	0.511 (0.047)	0.423 (0.051)	0.192 (0.229)	0.769 (0.501)
IDL-FC	0.586 (0.054)	0.539 (0.059)	0.525 (0.044)	0.494 (0.063)	0.44 (0.044)	0.402 (0.05)	0.19 (0.156)	0.33 (0.127)
IDL-L	NA	NA	0.57 (0.043)	NA	0.494 (0.048)	NA	0.148 (0.175)	0.444 (0.328)
IDL-P	0.566 (0.052)	0.536 (0.059)	0.575 (0.044)	0.488 (0.065)	0.434 (0.049)	0.412 (0.051)	0.153 (0.148)	0.292 (0.173)
IDL-PL	0.583 (0.052)	0.533 (0.058)	0.532 (0.045)	0.489 (0.064)	0.471 (0.047)	0.396 (0.05)	0.153 (0.18)	0.406 (0.184)
IDL-TG	0.603 (0.066)	0.595 (0.075)	0.658 (0.063)	0.567 (0.085)	0.432 (0.056)	0.315 (0.053)	0.11 (0.103)	0.047 (0.135)
HDL traits								
HDL-C	-0.117 (0.031)	-0.199 (0.045)	-0.136 (0.055)	-0.317 (0.052)	-0.045 (0.059)	-0.108 (0.05)	NA	NaN
ApoA1	-0.119 (0.042)	-0.193 (0.06)	0.023 (0.058)	-0.264 (0.071)	0.075 (0.064)	-0.13 (0.068)	-0.481 (0.271)	NA
HDL-D	-0.008 (0.027)	-0.124 (0.041)	0.004 (0.046)	-0.092 (0.048)	0.067 (0.045)	0.007 (0.041)	0.333 (0.114)	0.296 (0.1)
S-HDL-L	NA	NA	-0.098 (0.095)	NA	-0.037 (0.085)	NA	-0.312 (0.106)	-0.224 (0.087)
S-HDL-P	-0.265 (0.084)	-0.362 (0.113)	-0.13 (0.092)	-0.317 (0.119)	-0.053 (0.081)	-0.08 (0.094)	-0.331 (0.095)	-0.24 (0.083)
S-HDL-TG	0.354 (0.072)	0.386 (0.088)	0.65 (0.089)	0.475 (0.097)	0.351 (0.087)	0.283 (0.073)	0.253 (0.637)	-0.044 (0.466)
M-HDL-C	-0.323 (0.058)	-0.43 (0.079)	-0.364 (0.085)	-0.376 (0.091)	-0.46 (0.104)	-0.434 (0.075)	-0.508 (0.165)	-0.442 (0.143)
M-HDL-CE	-0.333 (0.058)	-0.458 (0.078)	-0.372 (0.09)	-0.385 (0.087)	-0.542 (0.105)	-0.443 (0.071)	-0.487 (0.157)	-0.413 (0.137)
M-HDL-FC	-0.275 (0.065)	-0.319 (0.08)	-0.262 (0.083)	-0.313 (0.092)	-0.313 (0.094)	-0.409 (0.082)	-0.649 (0.225)	-0.408 (0.166)
M-HDL-L	NA	NA	-0.311 (0.095)	NA	-0.474 (0.123)	NA	-0.606 (0.188)	-0.485 (0.155)
M-HDL-P	-0.298 (0.06)	-0.394 (0.086)	-0.273 (0.101)	-0.373 (0.1)	-0.565 (0.131)	-0.307 (0.079)	-0.694 (0.204)	-0.472 (0.166)
M-HDL-PL	-0.265 (0.058)	-0.346 (0.083)	-0.25 (0.09)	-0.335 (0.096)	-0.358 (0.104)	-0.3 (0.072)	-0.632 (0.191)	-0.486 (0.171)
L-HDL-C	-0.067 (0.03)	-0.144 (0.044)	-0.139 (0.051)	-0.144 (0.05)	-0.147 (0.052)	-0.049 (0.045)	0.516 (0.213)	0.575 (0.204)
L-HDL-CE	-0.063 (0.03)	-0.144 (0.044)	-0.116 (0.051)	-0.149 (0.051)	-0.134 (0.051)	-0.094 (0.047)	0.519 (0.23)	0.61 (0.206)
L-HDL-FC	-0.082 (0.03)	-0.144 (0.045)	-0.114 (0.053)	-0.128 (0.053)	-0.13 (0.051)	-0.03 (0.047)	0.518 (0.181)	0.59 (0.148)
L-HDL-L	NA	NA	-0.108 (0.05)	NA	-0.132 (0.052)	NA	0.457 (0.189)	0.541 (0.184)
L-HDL-P	-0.071 (0.028)	-0.146 (0.042)	-0.111 (0.05)	-0.13 (0.049)	-0.083 (0.05)	-0.1 (0.043)	0.422 (0.191)	0.476 (0.155)
L-HDL-PL	-0.087 (0.029)	-0.161 (0.043)	-0.141 (0.051)	-0.142 (0.051)	-0.105 (0.053)	-0.092 (0.044)	0.443 (0.202)	0.51 (0.169)
XL-HDL-C	0.055 (0.046)	-0.013 (0.068)	0.11 (0.066)	0.064 (0.073)	0.048 (0.069)	0.112 (0.068)	0.474 (0.223)	0.565 (0.196)
XL-HDL-CE	0.064 (0.044)	0.006 (0.066)	0.129 (0.066)	0.08 (0.07)	0.057 (0.068)	0.046 (0.075)	0.426 (0.177)	0.511 (0.206)
XL-HDL-FC	0.009 (0.039)	-0.05 (0.059)	0.066 (0.058)	-0.026 (0.067)	0.102 (0.06)	0.049 (0.066)	0.433 (0.16)	0.609 (0.159)
XL-HDL-L	NA	NA	0.073 (0.055)	NA	0.038 (0.058)	NA	0.358 (0.154)	0.481 (0.141)
XL-HDL-P	0.038 (0.033)	-0.022 (0.049)	0.112 (0.057)	0.017 (0.056)	0.083 (0.055)	0.023 (0.057)	0.41 (0.139)	0.39 (0.135)
XL-HDL-PL	0.029 (0.031)	-0.031 (0.046)	0.037 (0.05)	0.005 (0.055)	0.038 (0.052)	0.013 (0.046)	0.343 (0.118)	0.466 (0.12)
XL-HDL-TG	0.092 (0.027)	0.112 (0.041)	0.14 (0.047)	0.135 (0.047)	0.191 (0.042)	0.136 (0.039)	0.147 (0.074)	0.165 (0.086) Univariable MR results

**Appendix 3—table 3.** Mendelian randomization results using genome-wide significant SNPs and inverse variance weighted (IVW) estimator.

	Method: IVW + Significant SNPs					
Selection	GERA	GERA	GERA	GLGC	Davis	Kettunen
Exposure	Davis	Davis	Kettunen	Davis	Kettunen	Davis
Outcome	CAD	UKB	UKB	UKB	UKB	UKB
VLDL traits						
TG	0.184 (0.051)	0.278 (0.076)	NA	0.309 (0.074)	NA	0.207 (0.064)
VLDL-D	0.044 (0.06)	0.052 (0.09)	0.038 (0.102)	0.118 (0.091)	-0.083 (0.16)	-0.083 (0.138)
XS-VLDL-L	NA	NA	0.353 (0.08)	NA	0.372 (0.083)	NA
XS-VLDL-P	0.162 (0.04)	0.256 (0.059)	0.352 (0.081)	0.273 (0.063)	0.374 (0.084)	0.373 (0.095)
XS-VLDL-PL	0.165 (0.046)	0.262 (0.069)	0.37 (0.088)	0.27 (0.075)	0.443 (0.048)	0.401 (0.07)
XS-VLDL-TG	0.179 (0.041)	0.277 (0.061)	0.362 (0.082)	0.288 (0.062)	0.335 (0.076)	0.314 (0.08)
S-VLDL-C	0.237 (0.053)	0.343 (0.08)	NA	0.339 (0.083)	NA	0.443 (0.116)
S-VLDL-FC	0.21 (0.05)	0.307 (0.076)	0.344 (0.098)	0.314 (0.076)	0.262 (0.122)	0.397 (0.116)
S-VLDL-L	NA	NA	0.318 (0.095)	NA	0.27 (0.106)	NA
S-VLDL-P	0.188 (0.049)	0.274 (0.074)	0.311 (0.093)	0.29 (0.072)	0.266 (0.103)	0.331 (0.142)
S-VLDL-PL	0.198 (0.048)	0.291 (0.072)	0.342 (0.091)	0.3 (0.072)	0.281 (0.089)	0.331 (0.125)
S-VLDL-TG	0.174 (0.051)	0.255 (0.076)	0.296 (0.094)	0.28 (0.073)	0.261 (0.102)	0.262 (0.093)
M-VLDL-C	0.188 (0.053)	0.265 (0.08)	0.305 (0.096)	0.287 (0.077)	0.361 (0.078)	0.32 (0.134)
M-VLDL-CE	0.203 (0.051)	0.285 (0.077)	0.32 (0.098)	0.295 (0.076)	0.264 (0.094)	0.291 (0.125)
M-VLDL-FC	0.165 (0.056)	0.233 (0.084)	0.292 (0.098)	0.27 (0.08)	0.3 (0.084)	0.303 (0.104)
M-VLDL-L	NA	NA	0.265 (0.104)	NA	0.357 (0.096)	NA
M-VLDL-P	0.153 (0.056)	0.214 (0.085)	0.276 (0.104)	0.258 (0.081)	0.322 (0.092)	0.268 (0.074)
M-VLDL-PL	0.163 (0.054)	0.23 (0.082)	0.296 (0.097)	0.266 (0.078)	0.302 (0.084)	0.289 (0.095)
M-VLDL-TG	0.14 (0.058)	0.196 (0.087)	0.268 (0.107)	0.247 (0.083)	0.327 (0.093)	0.245 (0.091)
L-VLDL-C	0.177 (0.06)	0.24 (0.091)	0.288 (0.106)	0.286 (0.089)	0.108 (0.223)	0.31 (0.084)
L-VLDL-CE	0.178 (0.057)	0.245 (0.087)	0.262 (0.105)	0.279 (0.086)	0.182 (0.187)	0.299 (0.077)
L-VLDL-FC	0.176 (0.063)	0.242 (0.094)	0.295 (0.108)	0.298 (0.091)	0.321 (0.101)	0.314 (0.082)
L-VLDL-L	NA	NA	0.291 (0.119)	NA	0.125 (0.232)	NA
L-VLDL-P	0.164 (0.062)	0.227 (0.093)	0.269 (0.108)	0.275 (0.09)	0.332 (0.127)	0.247 (0.076)
L-VLDL-PL	0.173 (0.061)	0.23 (0.092)	0.308 (0.115)	0.284 (0.088)	0.32 (0.127)	0.302 (0.079)
L-VLDL-TG	0.149 (0.063)	0.202 (0.095)	0.268 (0.118)	0.267 (0.092)	0.33 (0.131)	0.302 (0.08)
XL-VLDL-L	NA	NA	0.263 (0.123)	NA	0.365 (0.286)	NA
XL-VLDL-P	0.149 (0.063)	0.206 (0.095)	0.247 (0.122)	0.268 (0.096)	0.346 (0.28)	0.245 (0.077)
XL-VLDL-PL	0.176 (0.067)	0.243 (0.101)	0.292 (0.119)	0.323 (0.101)	0.333 (0.265)	0.344 (0.133)
XL-VLDL-TG	0.151 (0.066)	0.205 (0.1)	0.241 (0.12)	0.282 (0.1)	0.323 (0.272)	0.249 (0.081)
XXL-VLDL-L	NA	NA	0.356 (0.127)	NA	-0.165 (0.425)	NA
XXL-VLDL-P	0.228 (0.067)	0.35 (0.099)	0.372 (0.119)	0.376 (0.098)	-0.12 (0.389)	0.006 (0.153)
XXL-VLDL-PL	0.211 (0.07)	0.31 (0.105)	0.275 (0.125)	0.399 (0.107)	-0.145 (0.395)	0.071 (0.191)
XXL-VLDL-TG	0.221 (0.067)	0.3 (0.102)	0.292 (0.126)	0.415 (0.104)	0.09 (0.36)	0.349 (0.303)
IDL/LDL traits						
LDL-C	0.427 (0.049)	0.431 (0.054)	0.409 (0.077)	0.409 (0.054)	0.416 (0.099)	0.422 (0.063)
ApoB	0.506 (0.058)	0.525 (0.065)	0.474 (0.093)	0.473 (0.064)	0.636 (0.092)	0.569 (0.071)
LDL-D	0.217 (0.151)	0.423 (0.161)	1.121 (0.178)	0.271 (0.143)	0.309 (0.126)	0.211 (0.081)
S-LDL-C	0.481 (0.056)	0.467 (0.063)	0.445 (0.087)	0.438 (0.063)	0.44 (0.128)	0.436 (0.076)
S-LDL-L	NA	NA	0.44 (0.09)	NA	0.456 (0.132)	NA
S-LDL-P	0.501 (0.059)	0.494 (0.068)	0.449 (0.093)	0.472 (0.067)	0.49 (0.139)	0.588 (0.097)
M-LDL-C	0.475 (0.057)	0.457 (0.064)	0.426 (0.08)	0.427 (0.064)	0.418 (0.111)	0.436 (0.087)
M-LDL-CE	0.485 (0.058)	0.47 (0.065)	0.432 (0.078)	0.436 (0.064)	0.43 (0.107)	0.444 (0.085)
M-LDL-L	NA	NA	0.43 (0.08)	NA	0.43 (0.11)	NA
M-LDL-P	0.479 (0.057)	0.465 (0.064)	0.437 (0.081)	0.44 (0.064)	0.413 (0.122)	0.439 (0.093)
M-LDL-PL	0.5 (0.063)	0.49 (0.071)	0.437 (0.087)	0.464 (0.07)	0.443 (0.132)	0.497 (0.099)
L-LDL-C	0.449 (0.055)	0.436 (0.061)	0.432 (0.076)	0.411 (0.061)	0.409 (0.106)	0.417 (0.076)
L-LDL-CE	0.464 (0.056)	0.451 (0.062)	0.426 (0.075)	0.422 (0.062)	0.416 (0.102)	0.433 (0.077)
L-LDL-FC	0.425 (0.054)	0.411 (0.059)	0.424 (0.074)	0.393 (0.059)	0.387 (0.105)	0.394 (0.078)
L-LDL-L	NA	NA	0.427 (0.074)	NA	0.407 (0.103)	NA
L-LDL-P	0.448 (0.054)	0.442 (0.06)	0.435 (0.075)	0.421 (0.059)	0.413 (0.104)	0.424 (0.075)
L-LDL-PL	0.444 (0.056)	0.438 (0.061)	0.441 (0.078)	0.423 (0.061)	0.42 (0.109)	0.429 (0.076)
IDL-C	0.447 (0.055)	0.455 (0.059)	0.451 (0.075)	0.433 (0.06)	0.439 (0.085)	0.422 (0.07)
IDL-FC	0.429 (0.055)	0.439 (0.059)	0.468 (0.075)	0.414 (0.059)	0.431 (0.081)	0.402 (0.074)
IDL-L	NA	NA	0.467 (0.075)	NA	0.445 (0.085)	NA
IDL-P	0.443 (0.055)	0.467 (0.06)	0.48 (0.077)	0.45 (0.059)	0.446 (0.088)	0.426 (0.071)
IDL-PL	0.429 (0.055)	0.443 (0.059)	0.473 (0.078)	0.427 (0.059)	0.435 (0.092)	0.407 (0.069)
IDL-TG	0.461 (0.07)	0.518 (0.076)	0.625 (0.098)	0.494 (0.073)	0.342 (0.085)	0.34 (0.123)
HDL traits						
HDL-C	-0.085 (0.044)	-0.156 (0.057)	-0.146 (0.085)	-0.195 (0.06)	-0.082 (0.159)	-0.015 (0.109)
ApoA1	-0.072 (0.054)	-0.155 (0.071)	-0.036 (0.09)	-0.194 (0.074)	0.001 (0.192)	0.066 (0.158)
HDL-D	-0.027 (0.042)	-0.071 (0.058)	-0.052 (0.073)	-0.092 (0.063)	0.073 (0.098)	0.074 (0.074)
S-HDL-L	NA	NA	-0.064 (0.148)	NA	-0.033 (0.092)	NA
S-HDL-P	-0.117 (0.087)	-0.172 (0.116)	-0.13 (0.146)	-0.298 (0.117)	-0.033 (0.09)	-0.115 (0.174)
S-HDL-TG	0.224 (0.063)	0.317 (0.082)	0.496 (0.107)	0.344 (0.085)	0.334 (0.096)	0.286 (0.17)
M-HDL-C	-0.214 (0.062)	-0.327 (0.078)	-0.48 (0.111)	-0.39 (0.079)	-0.423 (0.175)	-0.39 (0.159)
M-HDL-CE	-0.227 (0.062)	-0.338 (0.077)	-0.497 (0.111)	-0.4 (0.078)	-0.435 (0.194)	-0.341 (0.238)
M-HDL-FC	-0.158 (0.065)	-0.272 (0.084)	-0.341 (0.117)	-0.337 (0.085)	-0.288 (0.218)	-0.278 (0.144)
M-HDL-L	NA	NA	-0.436 (0.125)	NA	-0.514 (0.223)	NA
M-HDL-P	-0.172 (0.066)	-0.292 (0.087)	-0.414 (0.132)	-0.361 (0.089)	-0.386 (0.307)	-0.18 (0.118)
M-HDL-PL	-0.161 (0.064)	-0.275 (0.085)	-0.38 (0.126)	-0.345 (0.087)	-0.419 (0.301)	-0.2 (0.099)
L-HDL-C	-0.047 (0.044)	-0.097 (0.059)	-0.124 (0.08)	-0.133 (0.063)	0.022 (0.106)	0.021 (0.105)
L-HDL-CE	-0.049 (0.044)	-0.098 (0.059)	-0.12 (0.079)	-0.137 (0.063)	0.023 (0.112)	0.004 (0.106)
L-HDL-FC	-0.044 (0.046)	-0.094 (0.062)	-0.106 (0.082)	-0.127 (0.067)	0.038 (0.103)	0.017 (0.109)
L-HDL-L	NA	NA	-0.106 (0.077)	NA	0.034 (0.102)	NA
L-HDL-P	-0.045 (0.043)	-0.097 (0.058)	-0.102 (0.077)	-0.125 (0.063)	0.009 (0.111)	0.025 (0.11)
L-HDL-PL	-0.054 (0.044)	-0.11 (0.06)	-0.115 (0.079)	-0.14 (0.064)	0.006 (0.115)	0.016 (0.115)
XL-HDL-C	0.03 (0.06)	-0.012 (0.084)	0.014 (0.099)	-0.05 (0.088)	-0.015 (0.165)	0.161 (0.101)
XL-HDL-CE	0.03 (0.059)	-0.009 (0.081)	0.025 (0.098)	-0.042 (0.086)	-0.001 (0.166)	0.221 (0.107)
XL-HDL-FC	-0.003 (0.056)	-0.05 (0.076)	-0.001 (0.089)	-0.077 (0.081)	0.072 (0.11)	0.057 (0.092)
XL-HDL-L	NA	NA	0.001 (0.085)	NA	-0.009 (0.138)	NA
XL-HDL-P	0.015 (0.049)	-0.021 (0.067)	0.013 (0.088)	-0.042 (0.071)	0.103 (0.1)	0.135 (0.093)
XL-HDL-PL	0 (0.047)	-0.037 (0.065)	-0.026 (0.079)	-0.055 (0.069)	0.081 (0.088)	0.071 (0.069)
XL-HDL-TG	0.086 (0.041)	0.103 (0.059)	0.14 (0.075)	0.13 (0.063)	0.165 (0.043)	0.126 (0.051)

**Appendix 3—table 4.** Mendelian randomization results using genome-wide significant SNPs and the weighted median estimator.

	Method: Weighted median + Significant SNPs					
Selection	GERA	GERA	GERA	GLGC	Davis	Kettunen
Exposure	Davis	Davis	Kettunen	Davis	Kettunen	Davis
Outcome	CAD	UKB	UKB	UKB	UKB	UKB
VLDL traits						
TG	0.042 (0.055)	0.191 (0.072)	NA	0.228 (0.069)	NA	0.195 (0.077)
VLDL-D	-0.098 (0.052)	0.039 (0.095)	0.057 (0.11)	0.058 (0.093)	-0.107 (0.099)	-0.052 (0.115)
XS-VLDL-L	NA	NA	0.312 (0.076)	NA	0.393 (0.078)	NA
XS-VLDL-P	0.101 (0.037)	0.23 (0.052)	0.303 (0.079)	0.229 (0.052)	0.409 (0.08)	0.253 (0.059)
XS-VLDL-PL	0.096 (0.039)	0.242 (0.059)	0.352 (0.087)	0.228 (0.06)	0.422 (0.065)	0.319 (0.062)
XS-VLDL-TG	0.125 (0.041)	0.266 (0.057)	0.287 (0.079)	0.221 (0.056)	0.361 (0.084)	0.306 (0.069)
S-VLDL-C	0.187 (0.059)	0.232 (0.075)	NA	0.256 (0.074)	NA	0.303 (0.094)
S-VLDL-FC	0.152 (0.057)	0.207 (0.069)	0.289 (0.093)	0.227 (0.069)	0.316 (0.109)	0.279 (0.077)
S-VLDL-L	NA	NA	0.282 (0.083)	NA	0.306 (0.099)	NA
S-VLDL-P	0.131 (0.057)	0.202 (0.069)	0.275 (0.085)	0.221 (0.062)	0.291 (0.093)	0.226 (0.078)
S-VLDL-PL	0.137 (0.053)	0.205 (0.067)	0.283 (0.083)	0.218 (0.062)	0.305 (0.092)	0.263 (0.075)
S-VLDL-TG	0.112 (0.057)	0.204 (0.067)	0.216 (0.088)	0.229 (0.064)	0.267 (0.099)	0.244 (0.073)
M-VLDL-C	0.12 (0.058)	0.2 (0.07)	0.255 (0.088)	0.213 (0.066)	0.303 (0.099)	0.224 (0.081)
M-VLDL-CE	0.144 (0.054)	0.207 (0.071)	0.262 (0.087)	0.207 (0.068)	0.301 (0.098)	0.209 (0.072)
M-VLDL-FC	0.081 (0.058)	0.188 (0.074)	0.221 (0.087)	0.218 (0.068)	0.272 (0.102)	0.231 (0.08)
M-VLDL-L	NA	NA	0.227 (0.095)	NA	0.275 (0.109)	NA
M-VLDL-P	0.047 (0.06)	0.191 (0.072)	0.221 (0.096)	0.226 (0.069)	0.31 (0.104)	0.257 (0.079)
M-VLDL-PL	0.103 (0.056)	0.197 (0.071)	0.228 (0.089)	0.217 (0.064)	0.29 (0.104)	0.231 (0.078)
M-VLDL-TG	-0.005 (0.06)	0.199 (0.075)	0.224 (0.089)	0.222 (0.068)	0.318 (0.113)	0.233 (0.085)
L-VLDL-C	0.109 (0.068)	0.2 (0.078)	0.237 (0.093)	0.231 (0.075)	0.242 (0.122)	0.262 (0.088)
L-VLDL-CE	0.147 (0.063)	0.211 (0.079)	0.249 (0.09)	0.253 (0.073)	0.281 (0.11)	0.286 (0.081)
L-VLDL-FC	0.045 (0.065)	0.199 (0.085)	0.225 (0.093)	0.224 (0.077)	0.252 (0.125)	0.228 (0.089)
L-VLDL-L	NA	NA	0.243 (0.102)	NA	0.261 (0.122)	NA
L-VLDL-P	0.041 (0.064)	0.209 (0.082)	0.224 (0.092)	0.21 (0.079)	0.289 (0.122)	0.223 (0.086)
L-VLDL-PL	0.08 (0.063)	0.201 (0.08)	0.244 (0.101)	0.224 (0.077)	0.278 (0.123)	0.247 (0.092)
L-VLDL-TG	-0.008 (0.061)	0.215 (0.084)	0.225 (0.103)	0.161 (0.077)	0.286 (0.13)	0.277 (0.093)
XL-VLDL-L	NA	NA	0.262 (0.111)	NA	NA	NA
XL-VLDL-P	-0.026 (0.063)	0.207 (0.091)	0.289 (0.102)	0.192 (0.088)	NA	0.209 (0.101)
XL-VLDL-PL	-0.006 (0.067)	0.197 (0.094)	0.253 (0.094)	0.213 (0.088)	NA	0.24 (0.101)
XL-VLDL-TG	-0.026 (0.064)	0.214 (0.092)	0.229 (0.102)	0.191 (0.088)	NA	0.212 (0.099)
XXL-VLDL-L	NA	NA	0.316 (0.114)	NA	-0.156 (0.22)	NA
XXL-VLDL-P	0.091 (0.071)	0.236 (0.089)	0.267 (0.1)	0.263 (0.088)	-0.104 (0.173)	0.185 (0.098)
XXL-VLDL-PL	0.153 (0.082)	0.283 (0.096)	0.267 (0.11)	0.332 (0.095)	-0.139 (0.178)	0.126 (0.124)
XXL-VLDL-TG	0.126 (0.078)	0.266 (0.096)	0.244 (0.108)	0.339 (0.097)	0.227 (0.171)	0.23 (0.123)
IDL/LDL traits						
LDL-C	0.263 (0.053)	0.307 (0.066)	0.274 (0.05)	0.297 (0.063)	0.435 (0.072)	0.431 (0.067)
ApoB	0.365 (0.073)	0.472 (0.078)	0.381 (0.063)	0.375 (0.081)	0.624 (0.08)	0.565 (0.094)
LDL-D	0.306 (0.09)	0.413 (0.157)	0.467 (0.163)	0.271 (0.142)	0.294 (0.075)	0.193 (0.06)
S-LDL-C	0.271 (0.058)	0.342 (0.073)	0.343 (0.056)	0.273 (0.068)	0.498 (0.08)	0.274 (0.083)
S-LDL-L	NA	NA	0.354 (0.061)	NA	0.449 (0.081)	NA
S-LDL-P	0.355 (0.063)	0.366 (0.078)	0.397 (0.069)	0.329 (0.08)	0.49 (0.089)	0.581 (0.098)
M-LDL-C	0.283 (0.055)	0.313 (0.073)	0.299 (0.05)	0.244 (0.07)	0.474 (0.074)	0.297 (0.074)
M-LDL-CE	0.27 (0.055)	0.333 (0.077)	0.299 (0.051)	0.255 (0.071)	0.437 (0.081)	0.311 (0.077)
M-LDL-L	NA	NA	0.303 (0.053)	NA	0.432 (0.079)	NA
M-LDL-P	0.251 (0.057)	0.32 (0.071)	0.309 (0.054)	0.278 (0.07)	0.409 (0.072)	0.325 (0.078)
M-LDL-PL	0.343 (0.063)	0.337 (0.081)	0.316 (0.055)	0.318 (0.078)	0.457 (0.074)	0.353 (0.085)
L-LDL-C	0.251 (0.052)	0.29 (0.067)	0.303 (0.048)	0.231 (0.063)	0.45 (0.075)	0.309 (0.071)
L-LDL-CE	0.251 (0.054)	0.32 (0.068)	0.293 (0.052)	0.241 (0.066)	0.481 (0.074)	0.322 (0.077)
L-LDL-FC	0.251 (0.048)	0.214 (0.061)	0.301 (0.049)	0.214 (0.062)	0.427 (0.068)	0.289 (0.065)
L-LDL-L	NA	NA	0.289 (0.051)	NA	0.412 (0.07)	NA
L-LDL-P	0.281 (0.053)	0.321 (0.067)	0.29 (0.053)	0.244 (0.066)	0.42 (0.072)	0.351 (0.072)
L-LDL-PL	0.286 (0.05)	0.32 (0.067)	0.313 (0.052)	0.298 (0.065)	0.413 (0.074)	0.35 (0.076)
IDL-C	0.283 (0.056)	0.349 (0.068)	0.315 (0.053)	0.313 (0.07)	0.51 (0.072)	0.383 (0.068)
IDL-FC	0.283 (0.053)	0.334 (0.066)	0.337 (0.053)	0.314 (0.065)	0.422 (0.067)	0.367 (0.064)
IDL-L	NA	NA	0.329 (0.056)	NA	0.494 (0.069)	NA
IDL-P	0.331 (0.06)	0.44 (0.067)	0.343 (0.056)	0.371 (0.069)	0.463 (0.074)	0.328 (0.068)
IDL-PL	0.265 (0.055)	0.332 (0.066)	0.344 (0.056)	0.316 (0.066)	0.451 (0.072)	0.359 (0.066)
IDL-TG	0.233 (0.067)	0.371 (0.086)	0.605 (0.078)	0.337 (0.085)	0.315 (0.082)	0.215 (0.057)
HDL traits						
HDL-C	-0.017 (0.04)	-0.167 (0.058)	-0.17 (0.072)	-0.167 (0.058)	-0.096 (0.077)	-0.085 (0.07)
ApoA1	0.094 (0.049)	-0.06 (0.076)	-0.069 (0.087)	-0.167 (0.07)	0.005 (0.083)	-0.051 (0.121)
HDL-D	0.079 (0.034)	0.062 (0.061)	0.102 (0.064)	0.088 (0.061)	0.099 (0.061)	0.096 (0.058)
S-HDL-L	NA	NA	-0.174 (0.113)	NA	NA	NA
S-HDL-P	-0.173 (0.069)	0.018 (0.106)	-0.171 (0.109)	-0.235 (0.113)	NA	-0.049 (0.108)
S-HDL-TG	0.157 (0.061)	0.238 (0.085)	0.312 (0.105)	0.228 (0.086)	0.327 (0.105)	0.229 (0.076)
M-HDL-C	-0.169 (0.054)	-0.236 (0.082)	-0.264 (0.097)	-0.241 (0.077)	-0.392 (0.098)	-0.266 (0.084)
M-HDL-CE	-0.166 (0.053)	-0.23 (0.08)	-0.271 (0.099)	-0.238 (0.075)	-0.394 (0.103)	-0.23 (0.085)
M-HDL-FC	-0.166 (0.055)	-0.254 (0.086)	-0.281 (0.098)	-0.282 (0.087)	-0.28 (0.102)	-0.22 (0.1)
M-HDL-L	NA	NA	-0.296 (0.113)	NA	-0.448 (0.122)	NA
M-HDL-P	-0.157 (0.056)	-0.199 (0.09)	-0.298 (0.112)	-0.231 (0.086)	-0.291 (0.136)	-0.165 (0.131)
M-HDL-PL	-0.143 (0.058)	-0.183 (0.088)	-0.285 (0.108)	-0.183 (0.085)	-0.321 (0.114)	-0.203 (0.12)
L-HDL-C	0.086 (0.037)	-0.009 (0.066)	0.031 (0.083)	-0.032 (0.08)	0.003 (0.09)	0.006 (0.068)
L-HDL-CE	0.086 (0.038)	-0.011 (0.067)	0.075 (0.077)	-0.037 (0.076)	0.015 (0.091)	-0.006 (0.068)
L-HDL-FC	0.09 (0.039)	-0.005 (0.067)	0.079 (0.081)	-0.019 (0.076)	0.041 (0.078)	0.027 (0.074)
L-HDL-L	NA	NA	0.074 (0.077)	NA	0.068 (0.084)	NA
L-HDL-P	0.081 (0.036)	0.046 (0.062)	0.075 (0.074)	-0.01 (0.066)	0.066 (0.07)	0.078 (0.064)
L-HDL-PL	0.084 (0.039)	0 (0.067)	0.051 (0.082)	-0.021 (0.071)	0.054 (0.075)	0.074 (0.071)
XL-HDL-C	0.163 (0.047)	0.122 (0.091)	0.136 (0.087)	0.132 (0.09)	0.02 (0.098)	0.161 (0.096)
XL-HDL-CE	0.139 (0.044)	0.106 (0.088)	0.122 (0.09)	0.148 (0.085)	0.038 (0.091)	0.336 (0.092)
XL-HDL-FC	0.135 (0.048)	0.065 (0.079)	0.133 (0.081)	0.027 (0.077)	0.159 (0.079)	0.052 (0.086)
XL-HDL-L	NA	NA	0.119 (0.075)	NA	0.023 (0.078)	NA
XL-HDL-P	0.115 (0.035)	0.087 (0.07)	0.12 (0.073)	0.129 (0.067)	0.16 (0.071)	0.15 (0.073)
XL-HDL-PL	0.101 (0.037)	0.064 (0.07)	0.11 (0.072)	0.121 (0.069)	0.141 (0.069)	0.088 (0.065)
XL-HDL-TG	0.074 (0.027)	0.107 (0.047)	0.126 (0.051)	0.118 (0.042)	0.156 (0.05)	0.114 (0.045) Multivariable MR results

**Appendix 3—table 5.** Multivariable Mendelian randomization results (adjusted for HDL-C, LDL-C, and TG).

Trait	HDL-C	LDL-C	TG	Subfraction
VLDL traits				
VLDL-D	-0.251 (0.052)	0.29 (0.037)	0.6 (0.087)	-0.588 (0.094)
XS-VLDL-L	-0.086 (0.046)	0.286 (0.077)	0.089 (0.099)	0.132 (0.119)
XS-VLDL-P	-0.083 (0.045)	0.299 (0.078)	0.093 (0.106)	0.118 (0.125)
XS-VLDL-PL	-0.083 (0.046)	0.249 (0.098)	0.112 (0.076)	0.159 (0.12)
XS-VLDL-TG	-0.114 (0.046)	0.463 (0.079)	0.286 (0.173)	-0.157 (0.187)
S-VLDL-C	-0.267 (0.084)	0.754 (0.112)	1.033 (0.28)	-1.035 (0.323)
S-VLDL-FC	-0.195 (0.068)	0.898 (0.163)	0.935 (0.26)	-1.027 (0.337)
S-VLDL-L	-0.25 (0.072)	0.755 (0.112)	0.876 (0.233)	-0.898 (0.28)
S-VLDL-P	-0.31 (0.101)	0.819 (0.157)	1.209 (0.4)	-1.245 (0.463)
S-VLDL-PL	-0.168 (0.051)	0.673 (0.074)	0.626 (0.159)	-0.613 (0.182)
S-VLDL-TG	-0.499 (0.305)	0.906 (0.34)	2.532 (1.57)	-2.628 (1.741)
M-VLDL-C	-0.201 (0.068)	0.808 (0.127)	1.472 (0.424)	-1.433 (0.451)
M-VLDL-CE	-0.168 (0.061)	0.799 (0.111)	0.996 (0.249)	-1.035 (0.293)
M-VLDL-FC	-0.2 (0.072)	0.658 (0.089)	1.469 (0.417)	-1.412 (0.444)
M-VLDL-L	-0.355 (0.139)	0.602 (0.096)	1.787 (0.654)	-1.878 (0.75)
M-VLDL-P	-0.362 (0.124)	0.569 (0.08)	1.889 (0.676)	-1.974 (0.745)
M-VLDL-PL	-0.332 (0.141)	0.722 (0.159)	1.996 (0.869)	-2.012 (0.943)
M-VLDL-TG	-0.408 (0.153)	0.432 (0.061)	1.974 (0.772)	-2.133 (0.879)
L-VLDL-C	-0.216 (0.063)	0.509 (0.046)	1.163 (0.254)	-1.254 (0.297)
L-VLDL-CE	-0.272 (0.072)	0.465 (0.04)	1.038 (0.242)	-1.081 (0.282)
L-VLDL-FC	-0.144 (0.059)	0.493 (0.044)	1.233 (0.27)	-1.274 (0.308)
L-VLDL-L	-0.228 (0.066)	0.414 (0.045)	1.17 (0.263)	-1.277 (0.313)
L-VLDL-P	-0.115 (0.056)	0.442 (0.046)	1.351 (0.317)	-1.357 (0.344)
L-VLDL-PL	-0.221 (0.111)	0.473 (0.07)	2.135 (0.948)	-2.316 (1.112)
L-VLDL-TG	-0.196 (0.066)	0.355 (0.05)	1.357 (0.322)	-1.428 (0.372)
XL-VLDL-L	-0.126 (0.049)	0.451 (0.04)	0.896 (0.159)	-1.069 (0.203)
XL-VLDL-P	-0.127 (0.053)	0.474 (0.043)	1.038 (0.183)	-1.209 (0.238)
XL-VLDL-PL	-0.138 (0.055)	0.5 (0.044)	1.052 (0.204)	-1.214 (0.257)
XL-VLDL-TG	-0.129 (0.049)	0.424 (0.04)	0.944 (0.167)	-1.071 (0.205)
XXL-VLDL-L	-0.228 (0.067)	0.444 (0.043)	0.978 (0.207)	-1.355 (0.318)
XXL-VLDL-P	0.063 (0.076)	0.452 (0.05)	1.371 (0.384)	-1.639 (0.502)
XXL-VLDL-PL	-0.185 (0.056)	0.371 (0.042)	0.997 (0.185)	-1.259 (0.262)
XXL-VLDL-TG	-0.152 (0.059)	0.41 (0.04)	0.966 (0.19)	-1.202 (0.262)
LDL/IDL traits				
ApoB	-0.084 (0.046)	0.8 (0.146)	0.427 (0.101)	-0.532 (0.191)
LDL-D	-0.057 (0.042)	0.367 (0.03)	0.21 (0.053)	0.145 (0.061)
S-LDL-C	-0.062 (0.043)	0.614 (0.126)	0.261 (0.062)	-0.282 (0.152)
S-LDL-L	-0.06 (0.044)	0.584 (0.118)	0.266 (0.068)	-0.251 (0.145)
S-LDL-P	-0.033 (0.047)	0.589 (0.119)	0.29 (0.078)	-0.266 (0.151)
M-LDL-C	-0.082 (0.044)	0.623 (0.146)	0.203 (0.054)	-0.271 (0.162)
M-LDL-CE	-0.074 (0.043)	0.485 (0.167)	0.169 (0.059)	-0.088 (0.188)
M-LDL-L	-0.071 (0.044)	0.444 (0.171)	0.19 (0.063)	-0.069 (0.191)
M-LDL-P	-0.054 (0.044)	0.539 (0.153)	0.213 (0.063)	-0.179 (0.174)
M-LDL-PL	-0.081 (0.045)	0.747 (0.134)	0.232 (0.062)	-0.407 (0.162)
L-LDL-C	-0.071 (0.049)	0.437 (0.242)	0.167 (0.054)	-0.059 (0.261)
L-LDL-CE	-0.07 (0.048)	0.277 (0.301)	0.149 (0.065)	0.116 (0.321)
L-LDL-FC	-0.112 (0.057)	0.184 (0.304)	0.163 (0.053)	0.223 (0.315)
L-LDL-L	-0.075 (0.049)	0.229 (0.26)	0.146 (0.068)	0.167 (0.273)
L-LDL-P	-0.083 (0.046)	0.33 (0.2)	0.128 (0.064)	0.084 (0.213)
L-LDL-PL	-0.101 (0.046)	0.446 (0.177)	0.155 (0.057)	-0.036 (0.195)
IDL-C	-0.108 (0.057)	0.231 (0.215)	0.128 (0.064)	0.192 (0.229)
IDL-FC	-0.107 (0.05)	0.23 (0.147)	0.123 (0.056)	0.19 (0.156)
IDL-L	-0.1 (0.05)	0.274 (0.161)	0.123 (0.069)	0.148 (0.175)
IDL-P	-0.101 (0.047)	0.269 (0.134)	0.109 (0.071)	0.153 (0.148)
IDL-PL	-0.076 (0.048)	0.25 (0.162)	0.134 (0.071)	0.153 (0.18)
IDL-TG	-0.083 (0.046)	0.314 (0.069)	0.103 (0.089)	0.11 (0.103)
HDL traits				
ApoA1	0.345 (0.25)	0.544 (0.081)	0.334 (0.109)	-0.481 (0.271)
HDL-D	-0.442 (0.124)	0.421 (0.033)	0.111 (0.055)	0.333 (0.114)
S-HDL-L	-0.117 (0.046)	0.488 (0.044)	0.189 (0.054)	-0.312 (0.106)
S-HDL-P	-0.112 (0.046)	0.453 (0.035)	0.225 (0.056)	-0.331 (0.095)
S-HDL-TG	0.002 (0.145)	0.314 (0.156)	-0.007 (0.469)	0.253 (0.637)
M-HDL-C	0.179 (0.097)	0.36 (0.038)	0.147 (0.054)	-0.508 (0.165)
M-HDL-CE	0.167 (0.087)	0.319 (0.036)	0.166 (0.055)	-0.487 (0.157)
M-HDL-FC	0.339 (0.141)	0.436 (0.04)	0.247 (0.059)	-0.649 (0.225)
M-HDL-L	0.27 (0.108)	0.362 (0.032)	0.299 (0.063)	-0.606 (0.188)
M-HDL-P	0.302 (0.112)	0.386 (0.033)	0.371 (0.075)	-0.694 (0.204)
M-HDL-PL	0.311 (0.117)	0.402 (0.033)	0.333 (0.07)	-0.632 (0.191)
L-HDL-C	-0.589 (0.211)	0.469 (0.039)	0.146 (0.055)	0.516 (0.213)
L-HDL-CE	-0.602 (0.239)	0.477 (0.042)	0.137 (0.056)	0.519 (0.23)
L-HDL-FC	-0.573 (0.177)	0.437 (0.034)	0.171 (0.054)	0.518 (0.181)
L-HDL-L	-0.556 (0.193)	0.437 (0.034)	0.142 (0.055)	0.457 (0.189)
L-HDL-P	-0.515 (0.198)	0.417 (0.03)	0.133 (0.056)	0.422 (0.191)
L-HDL-PL	-0.53 (0.201)	0.415 (0.034)	0.152 (0.055)	0.443 (0.202)
XL-HDL-C	-0.447 (0.182)	0.342 (0.036)	0.071 (0.079)	0.474 (0.223)
XL-HDL-CE	-0.425 (0.146)	0.366 (0.038)	0.051 (0.069)	0.426 (0.177)
XL-HDL-FC	-0.459 (0.147)	0.377 (0.031)	0.097 (0.062)	0.433 (0.16)
XL-HDL-L	-0.405 (0.146)	0.364 (0.031)	0.077 (0.068)	0.358 (0.154)
XL-HDL-P	-0.451 (0.134)	0.374 (0.03)	0.078 (0.064)	0.41 (0.139)
XL-HDL-PL	-0.422 (0.119)	0.412 (0.033)	0.115 (0.055)	0.343 (0.118)
XL-HDL-TG	-0.186 (0.073)	0.336 (0.035)	0.045 (0.086)	0.147 (0.074)

**Appendix 3—table 6.** Multivariable Mendelian randomization results (adjusted for ApoA1, ApoB, and TG).

Trait	ApoA1	ApoB	TG	Subfraction
VLDL traits				
VLDL-D	-0.227 (0.067)	0.545 (0.092)	0.208 (0.139)	-0.32 (0.112)
XS-VLDL-L	-0.123 (0.063)	0.53 (0.163)	-0.121 (0.085)	0.084 (0.141)
XS-VLDL-P	-0.121 (0.064)	0.553 (0.17)	-0.123 (0.088)	0.061 (0.158)
XS-VLDL-PL	-0.147 (0.066)	0.273 (0.138)	0.028 (0.05)	0.253 (0.135)
XS-VLDL-TG	-0.102 (0.06)	0.762 (0.168)	0.069 (0.055)	-0.248 (0.15)
S-VLDL-C	-0.384 (0.141)	1.426 (0.354)	0.606 (0.351)	-1.265 (0.568)
S-VLDL-FC	-0.188 (0.077)	1.001 (0.235)	0.081 (0.053)	-0.489 (0.213)
S-VLDL-L	-0.46 (0.146)	1.776 (0.417)	0.7 (0.316)	-1.629 (0.586)
S-VLDL-P	-0.494 (0.159)	1.677 (0.386)	0.825 (0.372)	-1.644 (0.606)
S-VLDL-PL	-0.262 (0.097)	1.41 (0.343)	0.532 (0.261)	-1.213 (0.478)
S-VLDL-TG	-0.18 (0.069)	0.792 (0.121)	0.078 (0.051)	-0.301 (0.108)
M-VLDL-C	-0.157 (0.062)	0.867 (0.132)	0.085 (0.051)	-0.373 (0.118)
M-VLDL-CE	-0.221 (0.069)	1.224 (0.223)	0.47 (0.21)	-0.995 (0.338)
M-VLDL-FC	-0.222 (0.074)	0.902 (0.133)	0.482 (0.251)	-0.799 (0.311)
M-VLDL-L	-0.174 (0.065)	0.76 (0.104)	0.073 (0.05)	-0.298 (0.098)
M-VLDL-P	-0.181 (0.065)	0.764 (0.1)	0.077 (0.051)	-0.312 (0.096)
M-VLDL-PL	-0.159 (0.065)	0.776 (0.116)	0.08 (0.051)	-0.297 (0.106)
M-VLDL-TG	-0.263 (0.106)	0.724 (0.094)	0.547 (0.406)	-0.806 (0.455)
L-VLDL-C	-0.218 (0.084)	0.732 (0.101)	0.352 (0.278)	-0.609 (0.337)
L-VLDL-CE	-0.293 (0.079)	0.781 (0.096)	0.405 (0.189)	-0.673 (0.217)
L-VLDL-FC	-0.197 (0.069)	0.737 (0.094)	0.365 (0.25)	-0.619 (0.291)
L-VLDL-L	-0.194 (0.071)	0.666 (0.087)	0.289 (0.234)	-0.532 (0.278)
L-VLDL-P	-0.184 (0.061)	0.677 (0.086)	0.415 (0.217)	-0.617 (0.229)
L-VLDL-PL	-0.155 (0.063)	0.715 (0.095)	0.075 (0.051)	-0.287 (0.104)
L-VLDL-TG	-0.154 (0.062)	0.67 (0.083)	0.073 (0.05)	-0.252 (0.091)
XL-VLDL-L	-0.186 (0.066)	0.694 (0.088)	0.263 (0.19)	-0.577 (0.249)
XL-VLDL-P	-0.167 (0.061)	0.742 (0.088)	0.075 (0.05)	-0.373 (0.109)
XL-VLDL-PL	-0.191 (0.068)	0.712 (0.092)	0.271 (0.197)	-0.583 (0.268)
XL-VLDL-TG	-0.195 (0.068)	0.666 (0.087)	0.334 (0.21)	-0.603 (0.248)
XXL-VLDL-L	-0.173 (0.066)	0.732 (0.098)	0.088 (0.052)	-0.402 (0.144)
XXL-VLDL-P	-0.071 (0.065)	0.705 (0.097)	0.607 (0.321)	-1.089 (0.449)
XXL-VLDL-PL	-0.244 (0.082)	0.666 (0.091)	0.414 (0.257)	-0.814 (0.344)
XXL-VLDL-TG	-0.3 (0.091)	0.694 (0.095)	0.627 (0.306)	-1.075 (0.402)
IDL/LDL traits				
LDL-C	-0.119 (0.062)	0.247 (0.167)	0.066 (0.054)	0.319 (0.182)
LDL-D	-0.123 (0.06)	0.544 (0.091)	-0.036 (0.087)	0.119 (0.071)
S-LDL-C	-0.097 (0.06)	0.438 (0.216)	0.044 (0.051)	0.08 (0.238)
S-LDL-L	-0.097 (0.063)	0.503 (0.268)	0.043 (0.051)	-0.005 (0.29)
S-LDL-P	-0.059 (0.103)	0.932 (0.597)	-0.122 (0.112)	-0.362 (0.596)
M-LDL-C	-0.099 (0.065)	0.78 (1.034)	-0.172 (0.425)	-0.169 (0.909)
M-LDL-CE	-0.157 (0.128)	-0.346 (2.587)	0.195 (0.855)	0.854 (2.221)
M-LDL-L	-0.123 (0.095)	0.247 (1.479)	-0.001 (0.445)	0.32 (1.293)
M-LDL-P	-0.134 (0.07)	0.13 (0.286)	0.053 (0.052)	0.432 (0.31)
M-LDL-PL	-0.075 (0.077)	1.165 (0.868)	-0.248 (0.253)	-0.566 (0.839)
L-LDL-C	-0.855 (1.68)	-5.337 (13.402)	2.405 (5.735)	5.257 (11.72)
L-LDL-CE	-0.151 (0.065)	0.129 (0.193)	0.061 (0.052)	0.461 (0.213)
L-LDL-FC	-0.397 (0.219)	-1.139 (1.395)	0.786 (0.711)	1.531 (1.189)
L-LDL-L	-0.265 (0.148)	-0.854 (1.42)	0.41 (0.51)	1.266 (1.188)
L-LDL-P	-0.258 (0.153)	-0.607 (1.225)	0.276 (0.402)	1.064 (1.029)
L-LDL-PL	-0.312 (0.187)	-0.741 (1.411)	0.39 (0.518)	1.227 (1.245)
IDL-C	-0.3 (0.123)	-0.334 (0.616)	0.276 (0.254)	0.769 (0.501)
IDL-FC	-0.199 (0.069)	0.247 (0.118)	0.044 (0.049)	0.33 (0.127)
IDL-L	-0.215 (0.089)	0.021 (0.409)	0.101 (0.15)	0.444 (0.328)
IDL-P	-0.175 (0.075)	0.214 (0.172)	0.04 (0.051)	0.292 (0.173)
IDL-PL	-0.183 (0.07)	0.159 (0.172)	0.031 (0.049)	0.406 (0.184)
IDL-TG	-0.143 (0.075)	0.565 (0.146)	-0.119 (0.087)	0.047 (0.135)
HDL traits				
HDL-C	-1.513 (1.109)	0.982 (0.314)	0.27 (0.291)	1.446 (1.112)
HDL-D	-0.457 (0.138)	0.613 (0.073)	0.056 (0.049)	0.296 (0.1)
S-HDL-L	-0.128 (0.059)	0.524 (0.062)	0.067 (0.05)	-0.224 (0.087)
S-HDL-P	-0.132 (0.059)	0.531 (0.059)	0.071 (0.05)	-0.24 (0.083)
S-HDL-TG	-0.11 (0.113)	0.595 (0.221)	-0.057 (0.297)	-0.044 (0.466)
M-HDL-C	0.091 (0.084)	0.459 (0.101)	-0.1 (0.083)	-0.442 (0.143)
M-HDL-CE	0.09 (0.078)	0.291 (0.083)	0.082 (0.05)	-0.413 (0.137)
M-HDL-FC	0.148 (0.11)	0.378 (0.063)	0.066 (0.049)	-0.408 (0.166)
M-HDL-L	0.133 (0.091)	0.491 (0.097)	-0.029 (0.086)	-0.485 (0.155)
M-HDL-P	0.129 (0.097)	0.501 (0.097)	-0.004 (0.09)	-0.472 (0.166)
M-HDL-PL	0.162 (0.107)	0.519 (0.096)	-0.037 (0.087)	-0.486 (0.171)
L-HDL-C	-0.724 (0.232)	0.856 (0.132)	0.032 (0.093)	0.575 (0.204)
L-HDL-CE	-0.761 (0.236)	0.899 (0.145)	0.004 (0.084)	0.61 (0.206)
L-HDL-FC	-0.749 (0.174)	0.842 (0.102)	0.094 (0.05)	0.59 (0.148)
L-HDL-L	-0.717 (0.217)	0.815 (0.12)	0.023 (0.089)	0.541 (0.184)
L-HDL-P	-0.653 (0.191)	0.749 (0.104)	0.057 (0.049)	0.476 (0.155)
L-HDL-PL	-0.679 (0.201)	0.774 (0.109)	0.05 (0.049)	0.51 (0.169)
XL-HDL-C	-0.639 (0.194)	0.692 (0.095)	-0.058 (0.086)	0.565 (0.196)
XL-HDL-CE	-0.576 (0.2)	0.667 (0.096)	-0.077 (0.086)	0.511 (0.206)
XL-HDL-FC	-0.734 (0.174)	0.674 (0.073)	0.094 (0.052)	0.609 (0.159)
XL-HDL-L	-0.652 (0.168)	0.733 (0.097)	-0.06 (0.084)	0.481 (0.141)
XL-HDL-P	-0.52 (0.147)	0.691 (0.094)	-0.075 (0.084)	0.39 (0.135)
XL-HDL-PL	-0.652 (0.151)	0.687 (0.076)	0.079 (0.051)	0.466 (0.12)
XL-HDL-TG	-0.281 (0.111)	0.539 (0.09)	-0.152 (0.092)	0.165 (0.086) Q-statistics for multivariable Mendelian randomization Here we provide the list of modified Cochran's Q-statistics for the multivariable MR analyses (Appendix 3—tables 7 and 8).

**Appendix 3—table 7.** Modified Cochran’s Q-statistics (p-values) for the multivariable Mendelian randomization analyses (adjusted for HDL-C, LDL-C, and TG). DF is short for degrees of freedom.

Trait	DF	HDL-C	LDL-C	TG	Subfraction
VLDL traits					
VLDL-D	432	7640.8 (0)	1918.9 (7.9e-186)	877.6 (1.4e-32)	840.2 (1.6e-28)
XS-VLDL-L	436	7983.9 (0)	1104.9 (1.1e-59)	1935.8 (2.2e-187)	926 (1.9e-37)
XS-VLDL-P	436	7927.8 (0)	1066.6 (1.1e-54)	1814 (4.8e-167)	893.6 (9.6e-34)
XS-VLDL-PL	435	8291.5 (0)	968.1 (1.4e-42)	2771.5 (0)	849.8 (4.3e-29)
XS-VLDL-TG	431	7549.8 (0)	894.4 (1.3e-34)	739.5 (1.3e-18)	682.5 (1.2e-13)
S-VLDL-C	429	8598.1 (0)	652.6 (1.7e-11)	1220.7 (4.6e-77)	541.3 (0.00018)
S-VLDL-FC	434	7861.2 (0)	576 (5.4e-06)	519.4 (0.003)	507.9 (0.0082)
S-VLDL-L	438	7105.3 (0)	626 (8.5e-09)	525.2 (0.0026)	514.3 (0.0069)
S-VLDL-P	438	6686.5 (0)	616.5 (3.6e-08)	515.6 (0.0061)	507.3 (0.012)
S-VLDL-PL	437	7589.1 (0)	702.8 (1e-14)	591.5 (1.1e-06)	555.1 (0.00011)
S-VLDL-TG	437	7658.7 (0)	612.7 (5.3e-08)	498.9 (0.021)	494.5 (0.03)
M-VLDL-C	432	9167.8 (0)	740.8 (1.3e-18)	558.9 (3.5e-05)	551.5 (8.3e-05)
M-VLDL-CE	432	8055.2 (0)	705.9 (1.6e-15)	556.6 (4.6e-05)	539.7 (0.00031)
M-VLDL-FC	436	8272.8 (0)	814.8 (2.7e-25)	528.3 (0.0016)	519.1 (0.0037)
M-VLDL-L	429	7109.2 (0)	1269.2 (5.5e-84)	532.6 (0.00047)	515.9 (0.0025)
M-VLDL-P	436	8260.7 (0)	2059.5 (2.1e-208)	527.5 (0.0017)	516.8 (0.0046)
M-VLDL-PL	435	6849.2 (0)	599.6 (2.6e-07)	496.8 (0.021)	493.5 (0.027)
M-VLDL-TG	436	6123.7 (0)	9854.8 (0)	532.3 (0.0011)	521 (0.0031)
L-VLDL-C	435	8617.2 (0)	8966 (0)	654.7 (4.3e-11)	561.5 (3.9e-05)
L-VLDL-CE	434	6636.6 (0)	11134 (0)	581.6 (2.6e-06)	539.5 (0.00041)
L-VLDL-FC	431	7779.6 (0)	6691 (0)	595.1 (2.5e-07)	562.7 (1.9e-05)
L-VLDL-L	434	8104.9 (0)	5191.4 (0)	560.3 (3.9e-05)	548.6 (0.00015)
L-VLDL-P	435	2308 (5.1e-252)	10360.3 (0)	545.4 (0.00024)	537.9 (0.00054)
L-VLDL-PL	430	8155.4 (0)	1310.8 (8.6e-90)	491.8 (0.021)	489.7 (0.024)
L-VLDL-TG	438	8581.8 (0)	4800.1 (0)	569.1 (2.3e-05)	559.2 (7.5e-05)
XL-VLDL-L	437	8686.8 (0)	8322.2 (0)	674.7 (1.9e-12)	620.2 (1.7e-08)
XL-VLDL-P	431	8550.2 (0)	2459.4 (2e-280)	608.3 (3.6e-08)	588.6 (6.3e-07)
XL-VLDL-PL	431	7478.2 (0)	5042.5 (0)	613.3 (1.7e-08)	591.6 (4.1e-07)
XL-VLDL-TG	433	8237.3 (0)	9628.9 (0)	651.8 (4.6e-11)	618.3 (1.1e-08)
XXL-VLDL-L	439	8476.2 (0)	10436.4 (0)	652.9 (1.3e-10)	570.7 (2.2e-05)
XXL-VLDL-P	437	1291.3 (2.8e-85)	9987.4 (0)	540.3 (0.00053)	529.5 (0.0016)
XXL-VLDL-PL	436	9631.8 (0)	11287.1 (0)	641.6 (4.8e-10)	595.5 (5.3e-07)
XXL-VLDL-TG	429	7809.4 (0)	9476.4 (0)	595.6 (1.7e-07)	564 (1.2e-05)
LDL/IDL traits					
ApoB	435	9220.8 (0)	550.1 (0.00014)	1809.7 (1.2e-166)	535.1 (0.00072)
LDL-D	429	2909.2 (0)	3918.8 (0)	2706 (0)	1426.1 (2.9e-107)
S-LDL-C	431	8189.7 (0)	569.8 (7.8e-06)	4880.9 (0)	564.1 (1.6e-05)
S-LDL-L	435	8403.8 (0)	574.4 (7.8e-06)	3931.2 (0)	564.3 (2.7e-05)
S-LDL-P	431	7371.4 (0)	547.1 (0.00012)	3144.7 (0)	537.9 (0.00034)
M-LDL-C	430	9723.7 (0)	570.9 (5.8e-06)	6568.6 (0)	562.9 (1.6e-05)
M-LDL-CE	432	8442.1 (0)	558.3 (3.8e-05)	5773.6 (0)	549.1 (0.00011)
M-LDL-L	430	8801.7 (0)	555.4 (4e-05)	5176.1 (0)	548.2 (9.5e-05)
M-LDL-P	429	8798.9 (0)	541.6 (0.00018)	5049.7 (0)	535.2 (0.00035)
M-LDL-PL	436	7981.7 (0)	573.9 (9.6e-06)	4304.8 (0)	558.9 (6e-05)
L-LDL-C	432	8865.2 (0)	567.7 (1.2e-05)	6179.8 (0)	567 (1.3e-05)
L-LDL-CE	433	8464.3 (0)	558.7 (4.1e-05)	5731.3 (0)	555.6 (5.9e-05)
L-LDL-FC	431	7481.1 (0)	580.6 (1.9e-06)	6760.8 (0)	580.2 (2e-06)
L-LDL-L	433	8486.8 (0)	604.5 (8.9e-08)	5755.8 (0)	601.8 (1.3e-07)
L-LDL-P	434	8310.7 (0)	592.1 (6.3e-07)	5553.3 (0)	584.9 (1.7e-06)
L-LDL-PL	435	8341.4 (0)	588.5 (1.2e-06)	5327.8 (0)	577.4 (5.3e-06)
IDL-C	434	7873.9 (0)	645.5 (1.7e-10)	6336 (0)	642.1 (2.9e-10)
IDL-FC	432	8036 (0)	729.5 (1.4e-17)	6630.5 (0)	725.6 (3e-17)
IDL-L	434	7869.8 (0)	694.5 (2.4e-14)	5198.3 (0)	689 (7e-14)
IDL-P	436	9660.5 (0)	736.7 (9e-18)	5002 (0)	726.6 (7.1e-17)
IDL-PL	431	8432.6 (0)	680.6 (1.7e-13)	5023 (0)	677.4 (3e-13)
IDL-TG	436	7741.2 (0)	1077.5 (4.2e-56)	1992.9 (4.9e-197)	931.6 (4.4e-38)
HDL traits					
ApoA1	434	494.1 (0.024)	511.5 (0.006)	932.1 (1.8e-38)	492 (0.028)
HDL-D	438	783.5 (6.6e-22)	8500 (0)	5713.2 (0)	860.1 (9.4e-30)
S-HDL-L	438	3067.3 (0)	4414.6 (0)	3763.2 (0)	882.2 (3.7e-32)
S-HDL-P	438	2592.4 (1.1e-301)	7652.1 (0)	3097.3 (0)	951.1 (4.9e-40)
S-HDL-TG	425	896.9 (6.9e-36)	641.3 (5.2e-11)	540.1 (0.00013)	523 (8e-04)
M-HDL-C	437	957.6 (5.5e-41)	10172.4 (0)	4875.5 (0)	628.3 (4.9e-09)
M-HDL-CE	434	955.3 (3.2e-41)	1383.1 (1.7e-99)	4355.4 (0)	648.3 (1e-10)
M-HDL-FC	432	759.4 (2.4e-20)	2989.1 (0)	3512.2 (0)	538.2 (0.00037)
M-HDL-L	435	914.2 (3e-36)	11535.3 (0)	2327.7 (1.7e-255)	570.3 (1.3e-05)
M-HDL-P	434	997.6 (2.3e-46)	10709.6 (0)	1942.9 (3.2e-189)	561.3 (3.4e-05)
M-HDL-PL	434	977.8 (6.3e-44)	9439.9 (0)	2566 (1.8e-298)	581.3 (2.7e-06)
L-HDL-C	434	580 (3.2e-06)	1257.1 (4.4e-81)	4502.7 (0)	604.3 (1.1e-07)
L-HDL-CE	434	549 (0.00014)	930.2 (3e-38)	5517.2 (0)	557.2 (5.6e-05)
L-HDL-FC	441	627.6 (1.2e-08)	8415.3 (0)	3594 (0)	658.4 (7.9e-11)
L-HDL-L	434	603.6 (1.2e-07)	6743.8 (0)	5314.7 (0)	623.7 (5.7e-09)
L-HDL-P	432	601.1 (1.2e-07)	7769.3 (0)	6024.6 (0)	633.2 (8.6e-10)
L-HDL-PL	434	584.5 (1.8e-06)	9935.5 (0)	3544.3 (0)	611.3 (3.8e-08)
XL-HDL-C	430	732.9 (3.9e-18)	10426.6 (0)	2077.7 (1.4e-213)	686.9 (4e-14)
XL-HDL-CE	430	771.4 (9.3e-22)	8564.4 (0)	2457 (2.2e-280)	711.4 (3.3e-16)
XL-HDL-FC	432	761.8 (1.4e-20)	11265.2 (0)	2549.4 (3.1e-296)	770.9 (1.9e-21)
XL-HDL-L	429	767.6 (1.6e-21)	11490.7 (0)	2355.7 (1.2e-262)	784.6 (3.4e-23)
XL-HDL-P	433	724.9 (4.6e-17)	11372.5 (0)	2539.9 (3.9e-294)	798.5 (4.8e-24)
XL-HDL-PL	443	809.7 (7.8e-24)	10093.1 (0)	5762 (0)	895.4 (7.5e-33)
XL-HDL-TG	432	1849.1 (3.9e-174)	2635.9 (6.5e-312)	2240.8 (2.9e-241)	1267.8 (4.4e-83)

**Appendix 3—table 8.** Modified Cochran’s Q-statistics (p-values) for the multivariable Mendelian randomization analyses (adjusted for ApoA1, ApoB, and TG). DF is short for degrees of freedom.

Trait	DF	ApoA1	ApoB	TG	Subfraction
VLDL traits					
VLDL-D	297	1194.1 (9.1e-108)	550 (2.4e-17)	573.7 (8.2e-20)	606.7 (2.1e-23)
XS-VLDL-L	295	1185.1 (6.7e-107)	927 (2e-66)	1151.3 (2.2e-101)	887.9 (1.1e-60)
XS-VLDL-P	295	1194.9 (1.7e-108)	900 (1.9e-62)	895.5 (8.7e-62)	826.7 (6.4e-52)
XS-VLDL-PL	296	1148.5 (1.2e-100)	973.9 (3.2e-73)	2104.2 (1.4e-269)	961.4 (2.5e-71)
XS-VLDL-TG	302	1263.7 (1.1e-117)	757.9 (4.7e-41)	1308.1 (4.4e-125)	976.5 (4.6e-72)
S-VLDL-C	290	988.8 (4.4e-77)	394 (4.5e-05)	459.8 (7.8e-10)	402.6 (1.3e-05)
S-VLDL-FC	296	1092 (1.4e-91)	904 (8.6e-63)	1238.7 (2.1e-115)	1010.4 (8.1e-79)
S-VLDL-L	301	1107.9 (1.1e-92)	412.3 (2.1e-05)	420.8 (5.9e-06)	384.7 (0.00078)
S-VLDL-P	301	1116.6 (4.6e-94)	424.8 (3.3e-06)	401.3 (9.4e-05)	380.6 (0.0013)
S-VLDL-PL	299	1096 (2.3e-91)	428.9 (1.2e-06)	446 (7.1e-08)	432.1 (7.1e-07)
S-VLDL-TG	300	1152.4 (4.3e-100)	908.5 (1.8e-62)	1453.4 (1.8e-150)	1303.1 (7.1e-125)
M-VLDL-C	298	1171.2 (1e-103)	824 (7.3e-51)	1480 (8.9e-156)	1212.5 (1.8e-110)
M-VLDL-CE	298	1185.4 (4.9e-106)	564.4 (1.1e-18)	468.9 (9.2e-10)	431.6 (6.3e-07)
M-VLDL-FC	298	1190.4 (7.4e-107)	899.8 (1.1e-61)	415.2 (8.1e-06)	398.8 (8.4e-05)
M-VLDL-L	298	1144.1 (2.4e-99)	869.8 (2.4e-57)	1381 (1e-138)	1237.4 (1.4e-114)
M-VLDL-P	297	1121.3 (5.7e-96)	821.1 (1.1e-50)	1250.5 (4.6e-117)	1206.7 (8.1e-110)
M-VLDL-PL	298	1149.9 (2.8e-100)	843.2 (1.5e-53)	1391.8 (1.5e-140)	1226.3 (9.8e-113)
M-VLDL-TG	296	1187.4 (5.8e-107)	717.3 (5.8e-37)	366.3 (0.0033)	360.6 (0.006)
L-VLDL-C	295	1196.5 (9.1e-109)	820 (5.6e-51)	462.5 (1.5e-09)	376.9 (0.00088)
L-VLDL-CE	302	1183.1 (1.8e-104)	844.6 (7.4e-53)	541.8 (7.2e-16)	441.7 (2.6e-07)
L-VLDL-FC	295	1172.3 (8.2e-105)	851.6 (1.9e-55)	460.8 (2.1e-09)	406.2 (1.8e-05)
L-VLDL-L	295	1163.6 (2.2e-103)	797 (8.8e-48)	406.5 (1.7e-05)	391.5 (0.00014)
L-VLDL-P	293	1160.2 (2e-103)	809.5 (5.9e-50)	420.2 (1.5e-06)	407.9 (1e-05)
L-VLDL-PL	296	1292 (2.6e-124)	833.4 (1.3e-52)	1216.5 (9.7e-112)	1098.9 (1.1e-92)
L-VLDL-TG	294	1150.8 (1.3e-101)	1213.6 (7e-112)	1262.6 (5.2e-120)	1162.8 (1.5e-103)
XL-VLDL-L	294	1196 (5.4e-109)	829.4 (1.6e-52)	442 (4.9e-08)	423.6 (1.1e-06)
XL-VLDL-P	294	1265.9 (1.4e-120)	1180.9 (1.6e-106)	1202.2 (5.2e-110)	982.1 (5.4e-75)
XL-VLDL-PL	296	1199.1 (6.9e-109)	874.2 (1.9e-58)	421.2 (2.3e-06)	405.6 (2.3e-05)
XL-VLDL-TG	296	1184.3 (1.8e-106)	828.6 (5.9e-52)	430.8 (4.9e-07)	430.1 (5.5e-07)
XXL-VLDL-L	304	1119.2 (1.2e-93)	1041.9 (1.6e-81)	900.9 (2e-60)	699.6 (3.2e-33)
XXL-VLDL-P	303	1148 (1.7e-98)	876.4 (4e-57)	382.2 (0.0013)	366 (0.0076)
XXL-VLDL-PL	303	1203 (2.1e-107)	775.1 (4e-43)	438.1 (5.8e-07)	376.5 (0.0025)
XXL-VLDL-TG	303	1183 (3.7e-104)	881.8 (6.6e-58)	393.7 (0.00034)	372.7 (0.0039)
LDL/IDL traits					
LDL-C	293	1198.7 (9.6e-110)	938.8 (1.1e-68)	1060.2 (2.1e-87)	917.6 (1.5e-65)
LDL-D	296	1325.2 (6.7e-130)	747.9 (5.9e-41)	879.1 (3.7e-59)	1163.5 (4.6e-103)
S-LDL-C	296	1195.3 (2.9e-108)	706 (1.6e-35)	1426 (4.1e-147)	686.4 (4.8e-33)
S-LDL-L	296	1054.7 (1.1e-85)	608 (1e-23)	1519.6 (2.2e-163)	586.4 (2.5e-21)
S-LDL-P	297	852.9 (3.6e-55)	438.7 (1.6e-07)	954.7 (4.5e-70)	440.1 (1.3e-07)
M-LDL-C	296	1210.9 (8e-111)	396.2 (8.6e-05)	409 (1.4e-05)	398.9 (6e-05)
M-LDL-CE	295	1204.3 (4.8e-110)	350.8 (0.014)	361.7 (0.0048)	351.3 (0.013)
M-LDL-L	296	1212 (5.3e-111)	370 (0.0022)	392.3 (0.00015)	371.6 (0.0019)
M-LDL-P	297	1125.4 (1.2e-96)	623.9 (2.3e-25)	911.4 (1.3e-63)	582.4 (9.6e-21)
M-LDL-PL	299	1172.5 (1.2e-103)	399.3 (9.1e-05)	434.9 (4.5e-07)	396.2 (0.00014)
L-LDL-C	300	1174.6 (1.1e-103)	325.5 (0.15)	325.5 (0.15)	325.5 (0.15)
L-LDL-CE	299	1179.5 (9e-105)	769.8 (3e-43)	902.5 (7.7e-62)	743.8 (8.4e-40)
L-LDL-FC	295	1161 (5.8e-103)	322.4 (0.13)	323.2 (0.12)	322.3 (0.13)
L-LDL-L	300	1172.3 (2.6e-103)	336.9 (0.07)	349.6 (0.026)	340.3 (0.055)
L-LDL-P	300	1185.4 (2e-105)	352.1 (0.021)	378.4 (0.0014)	355.4 (0.015)
L-LDL-PL	296	1155.2 (9.8e-102)	343.2 (0.031)	360.1 (0.0063)	344.5 (0.027)
IDL-C	296	1181.7 (4.9e-106)	426.5 (9.8e-07)	427.6 (8.3e-07)	427.7 (8.1e-07)
IDL-FC	298	1096.5 (9.9e-92)	986.9 (1.1e-74)	1075.8 (1.9e-88)	975.4 (6.1e-73)
IDL-L	296	1176.1 (4e-105)	516.7 (3.3e-14)	531 (1.4e-15)	521.4 (1.2e-14)
IDL-P	297	1094.8 (9.5e-92)	910.9 (1.5e-63)	1103.9 (3.5e-93)	890.2 (1.6e-60)
IDL-PL	297	1107.8 (8.3e-94)	798.9 (1.3e-47)	931.6 (1.3e-66)	785.6 (8.6e-46)
IDL-TG	302	1060.8 (5.4e-85)	1052.1 (1.2e-83)	1092.6 (5.6e-90)	1118.3 (4.7e-94)
HDL traits					
HDL-C	298	318.7 (0.2)	336.3 (0.063)	329.1 (0.1)	318.6 (0.2)
HDL-D	300	637.4 (1.9e-26)	1156.6 (9.1e-101)	2305.2 (1.3e-305)	1183.8 (3.5e-105)
S-HDL-L	299	1597.7 (4.8e-176)	1222.5 (8.2e-112)	1916.4 (1.5e-233)	1057 (3.1e-85)
S-HDL-P	299	1666.8 (2.5e-188)	1249.4 (2.9e-116)	2146.5 (3.4e-276)	1103.3 (1.6e-92)
S-HDL-TG	299	899 (2.5e-61)	464.9 (2.4e-09)	464.5 (2.6e-09)	457.6 (9.2e-09)
M-HDL-C	299	1145.2 (3.2e-99)	768.2 (4.9e-43)	951.8 (4e-69)	786.8 (1.5e-45)
M-HDL-CE	299	1201.9 (2e-108)	1183.9 (1.7e-105)	2139.7 (6.4e-275)	843.9 (1.9e-53)
M-HDL-FC	298	881.1 (5.6e-59)	1252 (5.5e-117)	1989.1 (2.4e-247)	660.1 (1.8e-29)
M-HDL-L	299	1059 (1.5e-85)	766.4 (8.7e-43)	920.6 (1.7e-64)	672.5 (8.6e-31)
M-HDL-P	298	990.2 (3.5e-75)	760.4 (3.4e-42)	1027.6 (6.2e-81)	613.7 (4.7e-24)
M-HDL-PL	295	929.5 (8.3e-67)	763.9 (2.7e-43)	1057.2 (2.3e-86)	588.3 (1.1e-21)
L-HDL-C	299	579.3 (4.1e-20)	623.2 (5.7e-25)	639.6 (7.3e-27)	617.8 (2.3e-24)
L-HDL-CE	299	612.2 (1e-23)	650.7 (3.6e-28)	690.4 (5.5e-33)	644 (2.2e-27)
L-HDL-FC	308	581.7 (4.4e-19)	857.5 (2.6e-53)	1213.3 (1.4e-107)	915.8 (1.3e-61)
L-HDL-L	299	655.9 (8.7e-29)	747.7 (2.6e-40)	670.7 (1.4e-30)	713.2 (7.5e-36)
L-HDL-P	298	591.3 (1.5e-21)	934 (9.9e-67)	1269.7 (6.2e-120)	956.8 (3.9e-70)
L-HDL-PL	299	580 (3.4e-20)	863.5 (3.3e-56)	1262.4 (2.1e-118)	891.8 (2.8e-60)
XL-HDL-C	298	475.3 (2.7e-10)	734 (1e-38)	976.1 (4.9e-73)	554 (1.3e-17)
XL-HDL-CE	299	472.9 (5.4e-10)	736.9 (6.7e-39)	1117.4 (9e-95)	517.5 (6.5e-14)
XL-HDL-FC	295	527.8 (2.1e-15)	1182.8 (1.6e-106)	2169.4 (3.1e-282)	677.3 (4.3e-32)
XL-HDL-L	298	555.2 (9.6e-18)	701.2 (1.6e-34)	1014 (7.9e-79)	775.3 (3.4e-44)
XL-HDL-P	300	578.9 (6.3e-20)	744.5 (1.1e-39)	1015.5 (1.6e-78)	751.3 (1.4e-40)
XL-HDL-PL	306	604.9 (7.8e-22)	1153.9 (1.4e-98)	1899 (1.5e-227)	909.3 (3.7e-61)
XL-HDL-TG	300	702.2 (2.8e-34)	779.8 (2.2e-44)	1140.8 (3.2e-98)	1399.2 (3.7e-141)

## Appendix 4

Diagnostic plots and the genetic markers As mentioned above, RAPS is more robust against invalid instruments than other statistical methods for univariable MR, but it still needs the InSIDE assumption to be approximately satisfied. Zhao et al., 2019b described two diagnostic plots RAPS that checks whether there is clear evidence that the InSIDE assumption is violated. Here, we report these plots for HDL-C and M-HDL-P in different studies (Appendix 4—figures 1 and 2). Notice that a lack of evidence to falsify the InSIDE assumption does not mean that it is true. S-HDL-P M-HDL-P Genetic markers for M-HDL-P and S-HDL-P We can further assess the validity of the InSIDE assumption for M-HDL-P and S-HDL-P but examining the associations of their genetic instruments with the traditional lipid risk factors and other subfraction traits. We meta-analyzed the summary results in the two lipidome GWAS (Davis and Kettunen) and obtained SNPs that are associated with S-HDL-P and M-HDL-P (p-value $\leq 5\times 10^{-8}$; the results are LD-clumped). The next two Tables show some information about these genetic markers and their associations with other traits (Appendix 4—table 1 and 2). Appendix 4—figures 3 and 4 shows how adjusting for LDL-C and TG changes the effects of the selected SNPs for S-HDL-P and M-HDL-P on CAD. The adjusted effect on CAD is obtained by original effect on CAD – 0.45 * effect on LDL-C – 0.25 * effect on TG. After the adjustment, the associations of the genetic variants with CAD generally became closer to the fitted lines that correspond to the estimated effects of S-HDL-P and M-HDL-P.

**Appendix 4—table 1.** List of SNPs associated with M-HDL-P.

SNP	Chr	Gene	S-HDL-P	M-HDL-P	L-HDL-P	XL-HDL-P	HDL-C	LDL-C	TG	CAD
rs11208004	1	DOCK7	-0.039 **	-0.075 ***	-0.015	-0.002	-0.015 **	-0.050 ***	-0.069 ***	-0.012
rs4846913	1	GALNT2	-0.000	-0.061 ***	-0.062 ***	-0.023 .	-0.055 ***	-0.006	-0.044 ***	-0.025 .
rs2126259	8	LOC157273	-0.066 ***	-0.082 ***	-0.063 **	-0.025 .	-0.075 ***	-0.063 ***	-0.016 .	-0.004
rs2083637	8	LPL	-0.001	-0.058 ***	-0.092 ***	-0.053 **	-0.105 ***	-0.008	-0.108 ***	-0.047 **
rs10468017	15	ALDH1A2/LIPC	-0.096 ***	-0.060 ***	-0.209 ***	-0.202 ***	-0.118 ***	-0.002	-0.038 ***	-0.013
rs247616	16	CETP	-0.058 ***	-0.121 ***	-0.198 ***	-0.129 ***	-0.243 ***	-0.055 ***	-0.039 ***	-0.044 **
rs1943973	18	LIPG	-0.022	-0.108 ***	-0.104 ***	-0.078 ***	-0.077 ***	-0.024 **	-0.009	-0.016
rs737337	19	DOCK6	-0.047 .	-0.087 ***	-0.081 **	-0.058 *	-0.056 ***	-0.007	-0.011	-0.038 .
rs769449	19	APOE	-0.016	-0.078 ***	-0.071 ***	-0.015	-0.064 ***	-0.214 ***	-0.042 ***	-0.085 ***
rs7679	20	PCIF1/PLTP	-0.188 ***	-0.071 ***	-0.129 ***	-0.152 ***	-0.059 ***	-0.009	-0.051 ***	-0.025 .

**Appendix 4—table 2.** List of SNPs associated with S-HDL-P.

SNP	Chr	Gene	S-HDL-P	M-HDL-P	L-HDL-P	XL-HDL-P	HDL-C	LDL-C	TG	CAD
rs780094	2	GCKR	-0.074 ***	-0.034 *	-0.04 **	-0.034 *	-0.011 .	-0.021 **	-0.110 ***	-0.005
rs10935473	3	ST3GAL6-AS1	-0.052 ***	-0.014	-0.029 .	-0.031 *	-0.009 .	-0.003	-0.005	-0.007
rs4936363	11	SIK3	-0.064 ***	-0.046 **	-0.019	-0.006	-0.034 **	-0.018 .	-0.043 ***	-0.022
rs2043085	15	ALDH1A2/LIPC	-0.092 ***	-0.056 ***	-0.202 ***	-0.197 ***	-0.106 ***	-0.003	-0.033 ***	-0.008
rs1800588	15	ALDH1A2/LIPC	-0.106 ***	-0.050 **	-0.215 ***	-0.212 ***	-0.114 ***	-0.002	-0.044 ***	-0.015
rs289714	16	CETP	-0.077 ***	-0.122 ***	-0.162 ***	-0.102 ***	-0.214 ***	-0.036 ***	-0.035 ***	-0.012
rs6065904	20	PLTP	-0.171 ***	-0.060 ***	-0.127 ***	-0.149 ***	-0.052 ***	-0.008	-0.040 ***	-0.022 . Gene expression Here we provide evidence of variant-gene associations from Quantatitive Trait Locus (QTL) analyses in the GTEx project (Appendix 4—table 3).

**Appendix 4—table 3.** Tissue-specific gene expressions associated with the 4 discovered genetic markers in the GTEx project.

SNP.Id	Type	Gene.Symbol	Variant.Id	p value	Effect	Tissue
rs838880	eQTL	SCARB1	chr12_124777047_C_T_b38	1.5E-08	-0.20	Cells - Cultured fibroblasts
rs838880	sQTL	SCARB1	chr12_124777047_C_T_b38	4.1E-06	-0.34	Testis
rs737337	sQTL	DOCK6	chr19_11236817_T_C_b38	3.8E-43	0.99	Artery - Tibial
rs737337	sQTL	DOCK6	chr19_11236817_T_C_b38	6.4E-35	0.93	Adipose - Subcutaneous
rs737337	sQTL	DOCK6	chr19_11236817_T_C_b38	6.4E-35	0.93	Adipose - Subcutaneous
rs737337	sQTL	DOCK6	chr19_11236817_T_C_b38	1.6E-27	0.95	Esophagus - Muscularis
rs737337	sQTL	DOCK6	chr19_11236817_T_C_b38	3.2E-20	1.10	Colon - Sigmoid
rs737337	sQTL	DOCK6	chr19_11236817_T_C_b38	1.1E-17	0.93	Esophagus - Gastroesophageal Junction
rs737337	sQTL	DOCK6	chr19_11236817_T_C_b38	1.8E-09	0.81	Artery - Coronary
rs737337	sQTL	DOCK6	chr19_11236817_T_C_b38	1.2E-07	-0.49	Thyroid
rs737337	sQTL	KANK2	chr19_11236817_T_C_b38	4.4E-07	0.43	Artery - Tibial
rs737337	sQTL	KANK2	chr19_11236817_T_C_b38	3.5E-06	0.55	Heart - Left Ventricle
rs2943641	eQTL	IRS1	chr2_226229029_T_C_b38	1.4E-16	-0.30	Adipose - Subcutaneous
rs2943641	eQTL	IRS1	chr2_226229029_T_C_b38	6.1E-12	-0.23	Adipose - Visceral (Omentum)
rs2943641	eQTL	RP11-395N3.2	chr2_226229029_T_C_b38	3.5E-09	-0.23	Adipose - Subcutaneous
rs2943641	eQTL	RP11-395N3.1	chr2_226229029_T_C_b38	2.1E-07	-0.23	Adipose - Subcutaneous
rs2943641	eQTL	RP11-395N3.2	chr2_226229029_T_C_b38	2.3E-06	-0.19	Adipose - Visceral (Omentum)
rs6065904	eQTL	PLTP	chr20_45906012_G_A_b38	4.4E-22	-0.27	Muscle - Skeletal
rs6065904	eQTL	PLTP	chr20_45906012_G_A_b38	1.6E-16	-0.27	Adipose - Subcutaneous
rs6065904	eQTL	PLTP	chr20_45906012_G_A_b38	1.2E-15	-0.28	Adipose - Visceral (Omentum)
rs6065904	eQTL	PLTP	chr20_45906012_G_A_b38	3.2E-15	-0.42	Heart - Atrial Appendage
rs6065904	eQTL	PLTP	chr20_45906012_G_A_b38	7.2E-14	-0.25	Artery - Tibial
rs6065904	eQTL	PLTP	chr20_45906012_G_A_b38	1.8E-12	-0.27	Nerve - Tibial
rs6065904	eQTL	PLTP	chr20_45906012_G_A_b38	7.3E-12	-0.26	Esophagus - Muscularis
rs6065904	eQTL	PLTP	chr20_45906012_G_A_b38	2.0E-11	-0.29	Colon - Transverse
rs6065904	eQTL	PLTP	chr20_45906012_G_A_b38	4.1E-11	-0.32	Colon - Sigmoid
rs6065904	eQTL	PLTP	chr20_45906012_G_A_b38	1.2E-09	-0.26	Artery - Aorta
rs6065904	eQTL	PLTP	chr20_45906012_G_A_b38	4.2E-09	-0.29	Heart - Left Ventricle
rs6065904	eQTL	PLTP	chr20_45906012_G_A_b38	5.0E-09	-0.22	Thyroid
rs6065904	eQTL	PLTP	chr20_45906012_G_A_b38	1.7E-08	-0.29	Stomach
rs6065904	eQTL	PLTP	chr20_45906012_G_A_b38	4.3E-08	-0.24	Lung
rs6065904	eQTL	NEURL2	chr20_45906012_G_A_b38	6.6E-08	-0.26	Adipose - Subcutaneous
rs6065904	eQTL	PLTP	chr20_45906012_G_A_b38	6.8E-08	-0.33	Liver
rs6065904	eQTL	CTSA	chr20_45906012_G_A_b38	4.0E-07	-0.14	Nerve - Tibial
rs6065904	eQTL	PLTP	chr20_45906012_G_A_b38	5.3E-07	-0.37	Spleen
rs6065904	eQTL	NEURL2	chr20_45906012_G_A_b38	5.6E-07	-0.26	Adipose - Visceral (Omentum)
rs6065904	eQTL	PLTP	chr20_45906012_G_A_b38	8.9E-07	-0.46	Small Intestine - Terminal Ileum
rs6065904	eQTL	RP3-337O18.9	chr20_45906012_G_A_b38	1.8E-06	-0.22	Adipose - Subcutaneous
rs6065904	eQTL	WFDC3	chr20_45906012_G_A_b38	2.9E-06	-0.31	Nerve - Tibial
rs6065904	eQTL	DNTTIP1	chr20_45906012_G_A_b38	3.1E-06	-0.17	Artery - Tibial
rs6065904	eQTL	WFDC3	chr20_45906012_G_A_b38	4.5E-06	-0.27	Skin - Sun Exposed (Lower leg)
rs6065904	eQTL	SNX21	chr20_45906012_G_A_b38	4.8E-06	-0.15	Esophagus - Muscularis
rs6065904	eQTL	WFDC3	chr20_45906012_G_A_b38	8.9E-06	-0.27	Skin - Not Sun Exposed (Suprapubic)
rs6065904	eQTL	DNTTIP1	chr20_45906012_G_A_b38	1.0E-05	-0.14	Nerve - Tibial
rs6065904	eQTL	PLTP	chr20_45906012_G_A_b38	1.1E-05	-0.27	Prostate
rs6065904	eQTL	PLTP	chr20_45906012_G_A_b38	1.3E-05	-0.26	Pituitary
rs6065904	eQTL	PLTP	chr20_45906012_G_A_b38	1.4E-05	-0.21	Esophagus - Gastroesophageal Junction
rs6065904	eQTL	SNX21	chr20_45906012_G_A_b38	1.5E-05	-0.16	Esophagus - Mucosa
rs6065904	eQTL	SNX21	chr20_45906012_G_A_b38	1.7E-05	-0.23	Colon - Sigmoid
rs6065904	eQTL	SNX21	chr20_45906012_G_A_b38	1.7E-05	-0.17	Thyroid
rs6065904	eQTL	PLTP	chr20_45906012_G_A_b38	2.6E-05	-0.21	Breast - Mammary Tissue
rs6065904	eQTL	WFDC3	chr20_45906012_G_A_b38	2.9E-05	-0.23	Artery - Tibial
rs6065904	eQTL	NEURL2	chr20_45906012_G_A_b38	3.2E-05	-0.21	Thyroid
rs6065904	eQTL	PLTP	chr20_45906012_G_A_b38	3.7E-05	-0.17	Testis
rs6065904	eQTL	CTSA	chr20_45906012_G_A_b38	4.4E-05	-0.11	Skin - Not Sun Exposed (Suprapubic)
rs6065904	eQTL	WFDC3	chr20_45906012_G_A_b38	5.8E-05	-0.23	Muscle - Skeletal
rs6065904	eQTL	NEURL2	chr20_45906012_G_A_b38	8.2E-05	-0.27	Heart - Atrial Appendage
rs6065904	eQTL	SNX21	chr20_45906012_G_A_b38	8.4E-05	-0.17	Artery - Aorta
rs6065904	eQTL	NEURL2	chr20_45906012_G_A_b38	9.5E-05	-0.24	Artery - Aorta
rs6065904	eQTL	WFDC3	chr20_45906012_G_A_b38	9.5E-05	-0.31	Artery - Aorta
rs6065904	eQTL	RP3-337O18.9	chr20_45906012_G_A_b38	9.5E-05	-0.29	Heart - Atrial Appendage
rs6065904	eQTL	PLTP	chr20_45906012_G_A_b38	1.2E-04	-0.15	Skin - Sun Exposed (Lower leg)
rs6065904	eQTL	WFDC13	chr20_45906012_G_A_b38	1.5E-04	0.28	Esophagus - Muscularis
rs6065904	eQTL	DNTTIP1	chr20_45906012_G_A_b38	2.1E-04	-0.12	Cells - Cultured fibroblasts
rs6065904	sQTL	ZNF335	chr20_45906012_G_A_b38	3.3E-11	-0.65	Testis
rs6065904	sQTL	ACOT8	chr20_45906012_G_A_b38	1.3E-09	0.58	Heart - Left Ventricle
rs6065904	sQTL	PLTP	chr20_45906012_G_A_b38	4.5E-08	-0.32	Whole Blood
rs6065904	sQTL	PLTP	chr20_45906012_G_A_b38	4.8E-08	0.53	Spleen
rs6065904	sQTL	ACOT8	chr20_45906012_G_A_b38	1.3E-07	0.42	Esophagus - Mucosa
rs6065904	sQTL	ACOT8	chr20_45906012_G_A_b38	2.6E-07	0.49	Heart - Atrial Appendage
rs6065904	sQTL	CTSA	chr20_45906012_G_A_b38	1.0E-06	-0.41	Artery - Aorta
rs6065904	sQTL	ACOT8	chr20_45906012_G_A_b38	1.2E-06	0.33	Nerve - Tibial
rs6065904	sQTL	ACOT8	chr20_45906012_G_A_b38	1.2E-06	0.67	Brain - Spinal cord (cervical c-1)
rs6065904	sQTL	TNNC2	chr20_45906012_G_A_b38	2.1E-06	0.54	Brain - Cerebellum
rs6065904	sQTL	ACOT8	chr20_45906012_G_A_b38	2.1E-06	0.54	Brain - Cerebellum
rs6065904	sQTL	WFDC3	chr20_45906012_G_A_b38	5.5E-06	0.23	Skin - Sun Exposed (Lower leg)
rs6065904	sQTL	WFDC3	chr20_45906012_G_A_b38	9.4E-06	-0.28	Skin - Not Sun Exposed (Suprapubic)
